# QuantiFly: Robust Trainable Software for Automated *Drosophila* Egg Counting

**DOI:** 10.1371/journal.pone.0127659

**Published:** 2015-05-18

**Authors:** Dominic Waithe, Peter Rennert, Gabriel Brostow, Matthew D. W. Piper

**Affiliations:** 1 Department of Computer Science, University College London, Gower St, London, WC1E 6BT, United Kingdom; 2 Wolfson Imaging Centre, Weatherall Institute of Molecular Medicine, University of Oxford, John Radcliffe Hospital, Oxford, OX3 9DS, United Kingdom; 3 Institute of Healthy Ageing and Department of Genetics, Evolution and Environment, University College London, Gower St, London, WC1E 6BT, United Kingdom; Imperial College London, UNITED KINGDOM

## Abstract

We report the development and testing of software called QuantiFly: an automated tool to quantify *Drosophila* egg laying. Many laboratories count *Drosophila* eggs as a marker of fitness. The existing method requires laboratory researchers to count eggs manually while looking down a microscope. This technique is both time-consuming and tedious, especially when experiments require daily counts of hundreds of vials. The basis of the QuantiFly software is an algorithm which applies and improves upon an existing advanced pattern recognition and machine-learning routine. The accuracy of the baseline algorithm is additionally increased in this study through correction of bias observed in the algorithm output. The QuantiFly software, which includes the refined algorithm, has been designed to be immediately accessible to scientists through an intuitive and responsive user-friendly graphical interface. The software is also open-source, self-contained, has no dependencies and is easily installed (https://github.com/dwaithe/quantifly). Compared to manual egg counts made from digital images, QuantiFly achieved average accuracies of 94% and 85% for eggs laid on transparent (defined) and opaque (yeast-based) fly media. Thus, the software is capable of detecting experimental differences in most experimental situations. Significantly, the advanced feature recognition capabilities of the software proved to be robust to food surface artefacts like bubbles and crevices. The user experience involves image acquisition, algorithm training by labelling a subset of eggs in images of some of the vials, followed by a batch analysis mode in which new images are automatically assessed for egg numbers. Initial training typically requires approximately 10 minutes, while subsequent image evaluation by the software is performed in just a few seconds. Given the average time per vial for manual counting is approximately 40 seconds, our software introduces a timesaving advantage for experiments starting with as few as 20 vials. We also describe an optional acrylic box to be used as a digital camera mount and to provide controlled lighting during image acquisition which will guarantee the conditions used in this study.

## Introduction

The fruitfly *Drosophila melanogaster* is a widely used model organism for research. One of the most commonly measured metrics of adult physiology is female egg laying. Quantifying egg laying is a laborious and onerous task that requires the experimenter to score eggs using a dissecting microscope and a hand-held tally counter. Given the advances in computer vision and machine learning, we have developed an automated solution for this task.

In the imaging sciences, microscopy based experiments are often automated using software and custom macros which optimise acquisition and/or analysis of raw data [1–4]. So far, there is no analysis solution that can do this robustly and accurately for *Drosophila* eggs. Identifying and counting eggs automatically from digital images is a challenging problem due to a number of factors. For example, *Drosophila* may or may not lay their eggs uniformly across the surface of the food, resulting in clumping and eggs varying in orientation. Another significant challenge is that the food surface can often be cracked or rough, partially obscuring eggs because flies take advantage of these features when laying. Furthermore, different media formulations can optically change the appearance of the eggs. More intelligent and powerful computer vision (CV) algorithms, some of which mimic the human visual system, are now becoming available which can intepret even highly complex imagery [5]. These CV techniques often have a large number of parameters which require tuning for a given task (e.g. egg counting). Machine learning (ML) algorithms are often employed alongside CV algorithms because the ML component automates the parameter optimisation freeing the user from the task. The role of the user in this paradigm is to provide training material to the algorithm which allows the machine learning algorithm to refine the parameters accordingly. These algorithms are finding new application in the biosciences with many successful applications [6–8]. There is however still a lot of work to do to bring these techniques into the life sciences as these approaches require substantial validation in the biological domain as well as further attention applied to how user-friendly they are, and how they are distributed.

The goal of this study was to automate and optimize the task of counting eggs laid by female fruitflies. The standard manual technique is labor intensive and time consuming, meaning it is costly in terms of human resources, subject to user bias and fatigue errors. Manual counting takes between 30 s to 2 minutes per vial and a typical experiment will contain between 50–100 vials meaning that longitudinal studies of egg laying (e.g. [9]) require hundreds of hours committed to egg counting. Automation should enable experiments to be performed with greater ease and efficiency improving speed and reproducibility without accuracy tradeoffs. Here, we report the design and validation of a versatile and highly accurate piece of software that we call QuantiFly, that automates egg counting after a brief training period. This solution has the following features: 1) it is sufficiently quick and robust to compete with the existing manual method for accuracy and ease of use; 2) it is compatible with readily available digital capture equipment so that any laboratory can employ it without difficultly, and; 3) it is simple to deploy and interpret so that it is accessible to research scientists without computer science expertise. We additionally report the blueprints for a simple and easily fabricated physical apparatus for image acquisition to enhance speed, reproducibility and robustness and which can be used to replicate the results of this study.

## Materials and Methods

### Algorithm Implementation

The QuantiFly software utilises the density estimation approach first proposed in Lempitsky et al. (2010) [10] and later refined by Fiaschi et al (2012) [11]. We chose this approach because it is robust to changes in the appearance of eggs including those caused by clustering or altered orientation as well as overlooking irregularities in the egg laying surfaces. In this paradigm objects are represented as 2d gaussian probablility distributions with a distribution centred on each object in the scene. During the training phase the user is asked to click on the location of objects to be counted (in this case eggs), creating a sparse representation of instance locations within the image. The density value for pixels within the object are then generated through convolution of the grid with a gaussian of specific width (sigma). The sigma of the kernel should be chosen so that the output kernel is less than the size of the object, in this case (sigma = 1.0). Pixels close to an object will contain a density value which represents the probability of that pixel being the center of an egg. Summation of these pixel values for the whole image results in an overall count of eggs within the scene. Features for each pixel in the input image are then generated using basic image filters to decompose the scene into its visual components in a method which purportly mimics the mammalian visual system [12] by describing the structures within digital images at several scales of detail [13–15]. Subsequent to feature processing, each pixel in an input image is described by a feature vector, with each element of the vector corresponding to a different filter output. The features calculated within the QuantiFly software include Gaussian magnitude and derivative of Gaussian filters across multiple scales and also the minimum and maximum eigenvalues of a structural tensor calculated at each pixel location very similar to Fiaschi et al. [11]. In this paper we use as a feature desciptor with 5 different scales (sigma = 0.8, 1.6, 3.2, 6.4, 12.8) and apply it to the the red, green and blue channel of the input image, this technique is described throughout this paper as the ‘baseline’ approach.

The machine learning challenge for the density estimation approach is to organise and group pixel feature vectors and their output labels so that density interpretations can be predicted quickly and accurately for unlabelled input images. In this study, an ensemble of decision trees was used for solving this learning objective [16], in particular the Extremely Randomized Trees (ERT) framework [17]. The ERT framework takes the input image feature vectors and learns to associate them with output densities, which are supplied by the user during training. The ERT framework is highly accurate, but requires little or no optimisation when compared to other decision tree frameworks and so is less computationally demanding and faster to train [18]. Decision tree models utilise structures known as trees which are generated from the training data and represent a hierarchy of node splits. At each node of the tree, a random subset of features is chosen, along with a random threshold value for which to assess this feature between the minimum and maximum possible values. The population of input pixels at this node (which in the case of the top node, is all the training input pixels) is then used to assess which feature is most appropriate to parse the associated pixel output labels (in this case density values) for the population of input pixels. The goal of the algorithm is to pick the feature and associated threshold which reduces the entropy (variance in density labels) associated with the two output groups resulting from each node split. This process is repeated recursively until either the density values in a node are consistent or there are only a small number of values remaining at each node. The resulting structure is called a tree, at each level of the tree the input pixels are split into smaller and smaller groups. Tree generation is repeated multiple times (in this case 30 x) to produce an ensemble of decision trees. For an input image, the output class for each pixel is calculated from each tree in the ensemble, the average response from each tree for each pixel is then calculated and a density image generated. With this framework in place, new images can be processed, features calculated and output densities predicted for each pixel. To find the output egg count for a given image, all the output pixels are summed from the density image created from the ERT framework.

The QuantiFly software includes a novel training interface which allows the user to interactively train the system. In the results section it is shown how many labelled images are required to obtain a certain degree of accuracy, but from the outset it is informative and useful for the user who is training the system to see feedback in an iterative fashion to the training data, as they generate it. The user does this by selecting Regions of Interest (ROI) within the training image dataset and then provides labels for the location of each object (in this case the *Drosophila* eggs) by clicking on its position within this region. Once the system is trained the user can evaluate test images and obtain a prediction for the number of eggs present in this image. It is essential that the user mark the location of all the eggs within the training material, but with the ROI system it is possible to label just one region from an image and then evaluate the whole image and any other images using the available training data. This system allows users to gain a quick sense of how the software operates, before committing to larger scale training.

We found during this study that the accuracy of the original algorithm could be improved by applying a corrective transformation to the data. The parameters of the linear transform are established from minimsing the error resulting from comparing the number of labels for regions in the training set with their estimated number upon model evaluation:

$$\begin{array}{c} \hat{\beta },\hat{c}=\underset{\beta ,c}{argmin}\sum_{i}^{}\sum_{j}^{}\left(\beta PC_{ij}+c-AC_{ij}\right) \end{array}$$

Where *PC*_*ij*_ represents the predicted count estimated for each region (j) of each image (i) and ACij is the actual ground-truth count provided by the user for corresponding region and image. The gradient *β* and the offset c are calculated using the python NumPy polyfit function which performs the least-squares minimisation. Subsequently, the predicted counts of test images are corrected according to:

$$\begin{array}{c} CCi=\beta PC_{i}+c \end{array}$$

Where *CC*_*i*_ represents the corrected count for the image i.

### Software and Distribution

The QuantiFly software source code and binary distributions for linux, Mac and Windows are available to download (https://github.com/dwaithe/quantifly). The software was developed using python 2.7.8 [19]. The ERT learning framework was implemented using the scikit-learn python library (v 0.13.1) [20]. The training interface technology was developed using the python PyQT4 (v4.10.3) python library. The image features (gaussian gradient magnitude, laplacian of guassian, structure tensor eigenvalues) were calculated using the python bindings of the VIGRA image processing library [21]. Other essential python libraries used in the QuantiFly software include: SciPy (v0.14.0), NumPy (v1.8.2) and PyInstaller (v.2.1).

### Flies, media and egg laying

For all work, we used our laboratory strain of *Drosophila melanogaster* Dahomey. In general, 5 to 10 female flies were allowed to lay eggs on media in glass vials for up to 20 hours, at which point flies were transferred to new media. The vials were then counted immediately or frozen at -20 C for counting at a later time. The eggs in each vial were counted using a dissecting microscope and hand-held tally counter. The time taken to process a batch of vials was recorded. A photo of each of the vials was then captured using a Logitech c920 webcam mounted on an adjustable stand or in the camera-housing enclosure described below. For acquisition we used the linux GTK+ UVC viewer software, although the default software for any webcam would be suitable. All media designated as opaque is sugar/yeast (SY) food, which was prepared at 1x concentration (SYBrewers in Bass et al [22]) and the translucent media correspond to the holidic, or defined, medium (DM) described in Piper et al [23].

### Digital ground-truths and accuracy calculation

To assess the performance of different algorithms used in this study and to train the system in a reproducible way, digital ground-truth data was required in addition to the conventional manual assessment of egg counts in each image. Vial images were imported into Fiji one at a time and then annotated using the point tool [2]. Annotated images were then exported as binary images and then imported with an evaluation script to train the algorithm and to evaluate its performance. Throughout this study the accuracy of the algorithms are evaluated as percent accuracy, which represents the absolute difference of the ground-truth and the predicted count over the ground-truth count:

$$\begin{array}{c} Accuracy\left(\%\right)=\frac{1}{n}\sum_{i}^{n}\left(|PC_{i}-GT_{i}|/GT_{i}\right).100 \end{array}$$

where n is the total number of images in the dataset and GT represents the ground truth count for each image. The package GraphPad Prism (v6,0d) was used for performing the statistics in this study. The vial images and ground-truth annotations used in this study are available through the software website (https://github.com/dwaithe/quantifly).

### Camera Housing

In order to assist the long-term use of QuantiFly by a variety of users, we have also constructed a camera-housing box consisting of a mounted web-cam, physical guides to control the vial position in the centre of the image and an array of LEDs to provide consistent and repeatable illumination of the food surface from above for opaque media and underneath for transparent media. Both image acquisition and control over illumination is achieved by an in-built arduino chip connected to a computer. Please enquire for the blueprints. The camera-housing box was developed in collaboration with, and manufactured by Luis Garcia of Polygonal Tree (http://polygonaltree.github.io/egg_counter).

## Results and Discussion

### Core Algorithm

**Fig 1:**
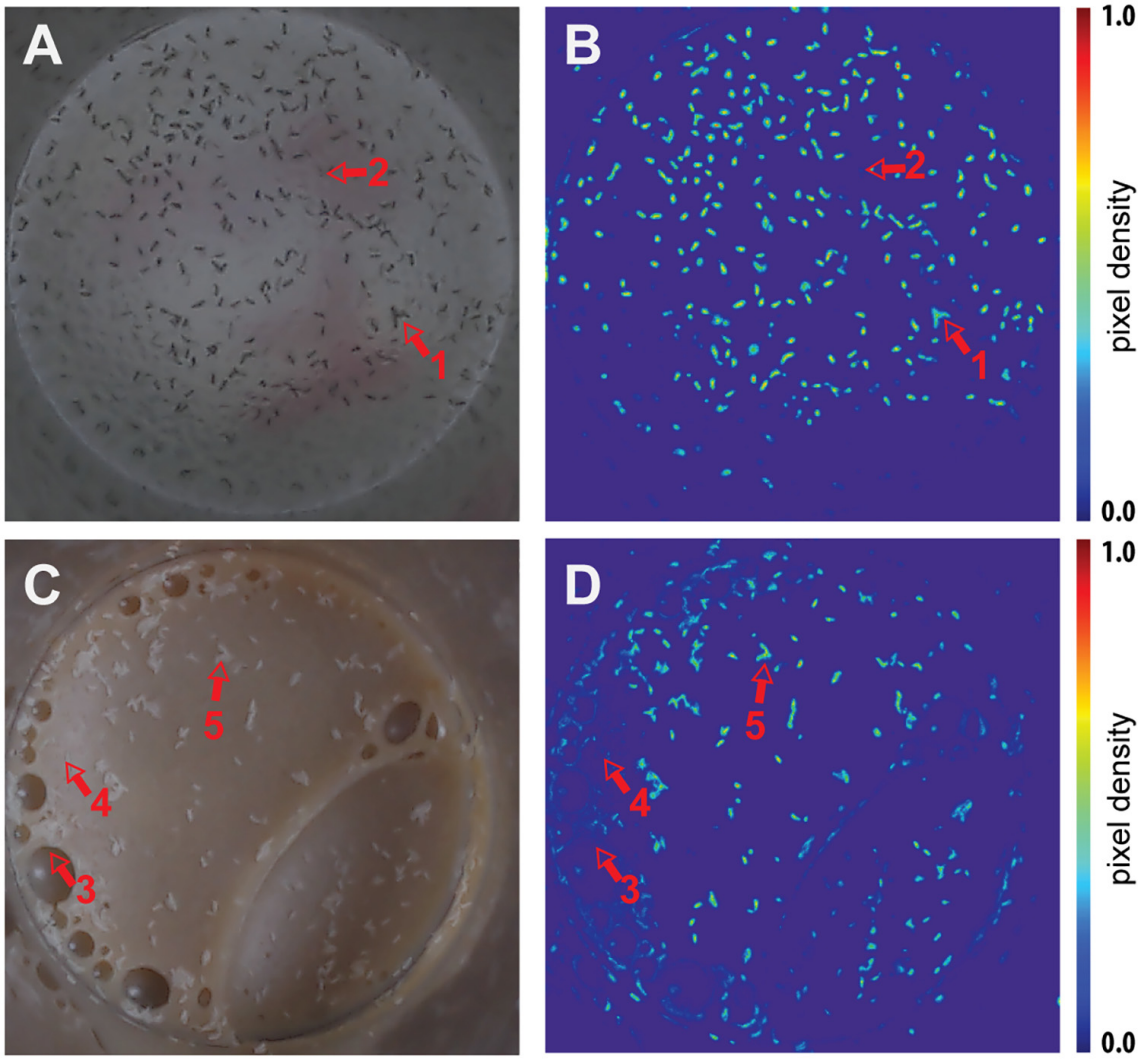
Typical vial images illustrating varying lighting conditions and irregularities in media surface. Original vial image (A) and egg density representation from QuantiFly (B) for transparent defined medium (DM). Original vial image (C) and egg density representation (D) for opaque sugar/yeast (SY) medium. Red arrows highlight artefacts present in images: (1) clumped eggs; (2) marks on vial base; (3) bubble artefacts in media; (4) specular reflection from vial surface, and; (5) clumped eggs on food surface. Colour bar represents pixel density estimate of an egg.

Due to the non-trivial nature of the imaging and counting problems associated with automating fly egg counting, we used an advanced computer vision and machine learning strategy using a density estimation model [10, 11]. As an initial check of the appropriateness of this technique, we trained the baseline algorithm by exhaustively labelling eggs in an image of eggs laid on transparent fly food and then used the trained algorithm to evaluate the same image, the procedure was then repeated for eggs laid on opaque fly food. Visually comparing the algorithms density output (Fig 1B and 1D) with the original image (Fig 1A and 1C) showed the eggs were well represented as areas of higher density, even when they varied in orientation, intensity and some were found in clusters. Furthermore, the density estimation approach is tolerant to a number of visual artefacts that appear in vial images, such as marks on the container that appear in the periphery of the images, reflected images of eggs, and bubbles in the medium surface (Fig 1).

An inherent feature of using an extremely random decision tree framework for learning is that training involves a degree of randomness such that for repeated rounds of algorithm training on a labelled image, a slightly different learned model is created. Subsequently, when these models are directed to assess the number of eggs in a new image, each will yield a slightly different count. Once trained however, a single ensemble model becomes fixed and it will always evaluate an image in the same way. To assess the effect of this variation on our final count data, we took a single image of eggs on translucent fly medium, exhaustively labeled the eggs and trained 100 x naïve models on the labeled image. The distribution of derived counts was broadly Gaussian, generating an average prediction of 214.8 ± 15.4 (s.d.), which was an accurate representation of the ground truth count of 213. Thus the variation introduced into counting by the variation in model training is less than 10% of the actual ground truth number for a single training image. This is a small amount given the expected biological variation and with additional training images the variation drops further (e.g with 5 images s.d. is 3%).

### Addressing Bias in Algorithm Predictions

**Fig 2:**
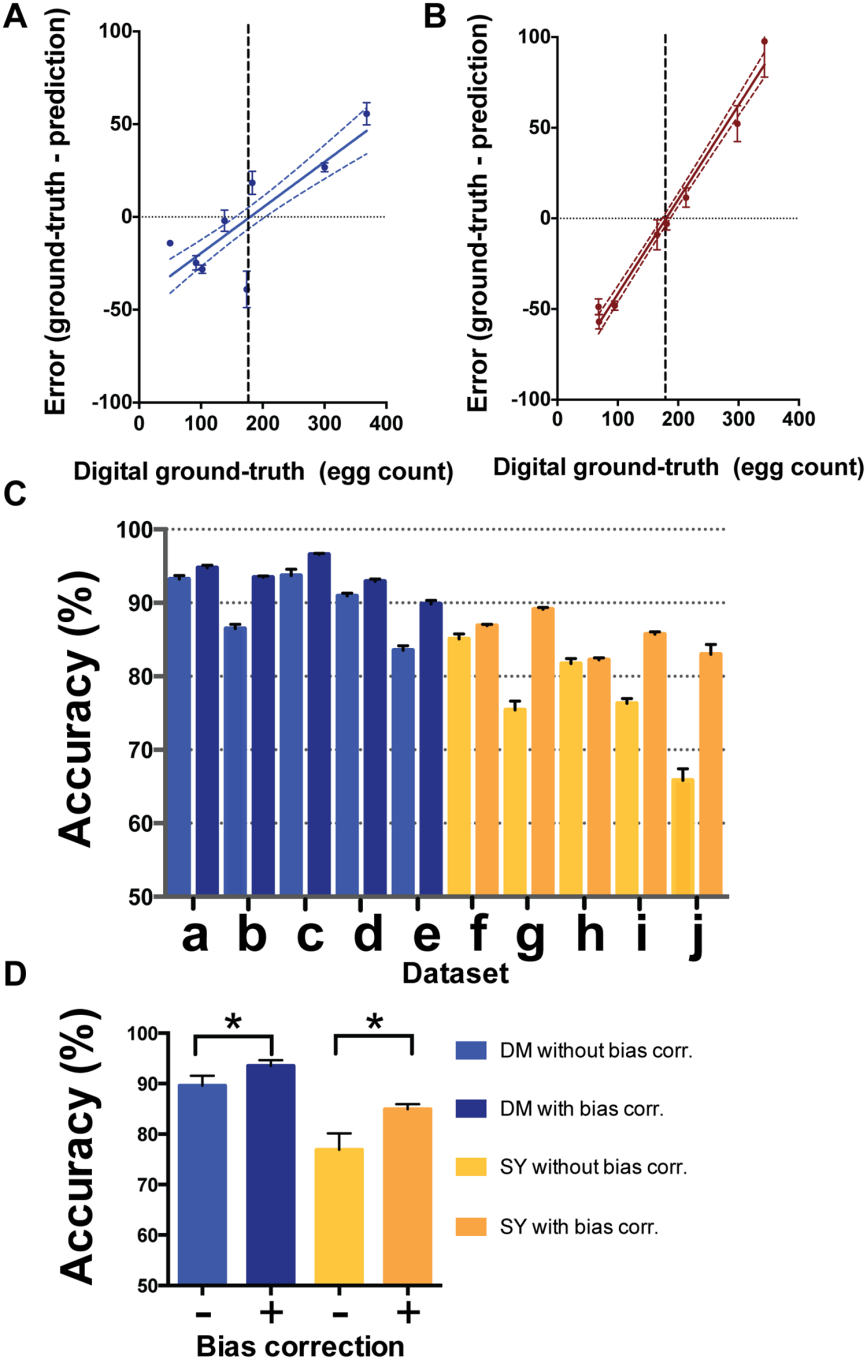
Bias in baseline algorithm predictions can be corrected using linear transformation. The baseline algorithm tends to over-estimate low egg counts and under-estimate high egg counts with the error polarity and magnitude related to the mean of the training set. (A-B) error associated with egg counts (y-axis) from a set of vials containing transparent (A) or opaque (B) media selected to span a broad range of ground-truth values (x-axis). There is a significant linear correlation with an x-axis intercept close to the mean of the distribution in these two datasets, confidence interval (95%) is indicated as dashed coloured lines. (C) Through calculating bias correction during training it is possible to correct the baseline predictive estimate for vial counts. The accuracy was improved for 10 different datasets when bias correction was applied, depicted in panel (transparent media; a-e) and (opaque media; f-j). (D) Summary comparison of transparent media datasets without correction (light-blue) and with bias correction (dark-blue) and of opaque media without correction (yellow-orange) and with correction (dark-orange). Bias correction increased accuracy in both media types. Each dataset was captured independently and contains 8 vial images with the exception of e and j which represent the vials depicted in A and B repectively. A Leave-one-out cross-validation strategy was performed for each vial (7 in and 1 out). Accuracy represents average of five statistical replicates, error bars represent SE (n = 5, statistical replicates); * represents P<0.05, Mann-Whitney one-tailed test.

We next asked how sensitive is the algorithm to the range of egg densities experienced during training. To do this, we selected images of 8 vials with translucent media and egg numbers ranging in density from ∼15 to ∼410 eggs per vial. After training the algorithm on each of these images, we assessed its ability to retrieve the number of eggs in the same 8 vials (Fig 2; also see S1 Fig for all raw images used to generate all data in this manuscript). We noticed that for a given vial, the predictive accuracy of the baseline algorithm worsened as the corresponding ground truth for that vial deviated more from the mean of the eight vials used for training (Fig 2A) such that those with ground-truths less than the mean tended to be over-estimated and those with ground-truths above the mean tended to be under-estimated. This effect was also apparent when we repeated the analysis on eight vials containing opaque medium (Fig 2B). For both media types, there was a strong positive linear correlation between the prediction error and the vial ground-truth count (p < 0.001, linear regression t-test) intersecting the x-axis at the mean of the ground-truth counts for the 8 vials. The bias-variance dilemma is a well-known phenomenon in machine learning and is known to exist in the extremely random decision tree framework [17, 24]. Bias results from using a model and/or features which are not perfectly suited to the data being fit. In this case we believe that the bias stems from slight insufficiences in the feature detector, in its ability to discriminate image features that are from eggs partially occluded in clusters. The feature vector although still correlated with density becomes diluted, and so pixels from high density areas will become reduced and pixels from low density areas will become eleviated. Fortunately, the predictable nature of this bias means that knowledge from the training data can be used to apply corrections to the output counts to enhance their accuracy.

We assessed the performance of this simple adjustment by using 7 of the 8 images for training and bias calculation and then evaluating the performance on the remaining 8th image, this was repeated for each image in the dataset. This approach was applied to 8 different datasets (a-d, f-j) comprising of independent vial images and datasets (e and j) which represent the data in Fig 2A–2B respectively. The bias correction improved the algorithm performance for estimating the true egg counts (Fig 2C) as well as improving the accuracy of the group average (for all the transparent and opaque media datasets) from 89.6% to 93.5% and 76.9% to 85.0% for the transparent and opaque media respectively (Fig 2D; p<0.01, Wilcoxon 1-tailed pair-wise test) (Table 1).

**Table 1:** Performance of algorithm on defined media and SY-media datasets.

Media:	Defined Media (transparent)				
Dataset:	a	b	c	d	e
Number of vials:	8	8	8	8	8
Number of eggs:	848	1847	1534	505	1407
Baseline Accuracy:	93.5 ± 0.5%	86.5 ± 0.6%	93.7 ± 0.8%	91.0 ± 0.4%	83.5 ± 0.6%
QuantiFly Accuracy:	94.8 ± 0.3%	93.5 ± 0.2%	96.6 ± 0.1%	92.9 ± 0.3%	89.8 ± 0.5%
Media:	SY Media (opaque)				
Dataset:	f	g	h	i	j
Number of vials:	8	8	8	8	8
Number of eggs:	1119	671	720	1533	1432
Baseline Accuracy:	85.1 ± 0.7%	75.4 ± 1.2%	81.7 ± 0.7%	76.3 ± 0.7%	65.9 ± 0.7%
QuantiFly Accuracy:	86.9 ± 0.2%	89.1 ± 0.3%	82.2 ± 0.3%	85.7 ± 0.3%	83.0 ± 0.6%

Datasets contain 8 vial images. A Leave-one-out cross-validation strategy was performed for each vial (7 in and 1 out). Standard error was generated from n = 5 trials (statistical replicates). QuantiFly Accuracy represents the baseline algorithm with the bias correction.

### Introducing QuantiFly Software

To implement the described technique in a user-friendly form, we developed a software package called QuantiFly. This consists of the baseline algorithm with the bias correction, along with a novel ROI labelling interface. Although the software can in principle be tuned to any counting task, such as cells in microscopy images or microbial colonies on plates, the parameters and user interface of our software have been developed for the task of *Drosophila* egg counting. A full user manual is available in S1 Text.

**Fig 3:**
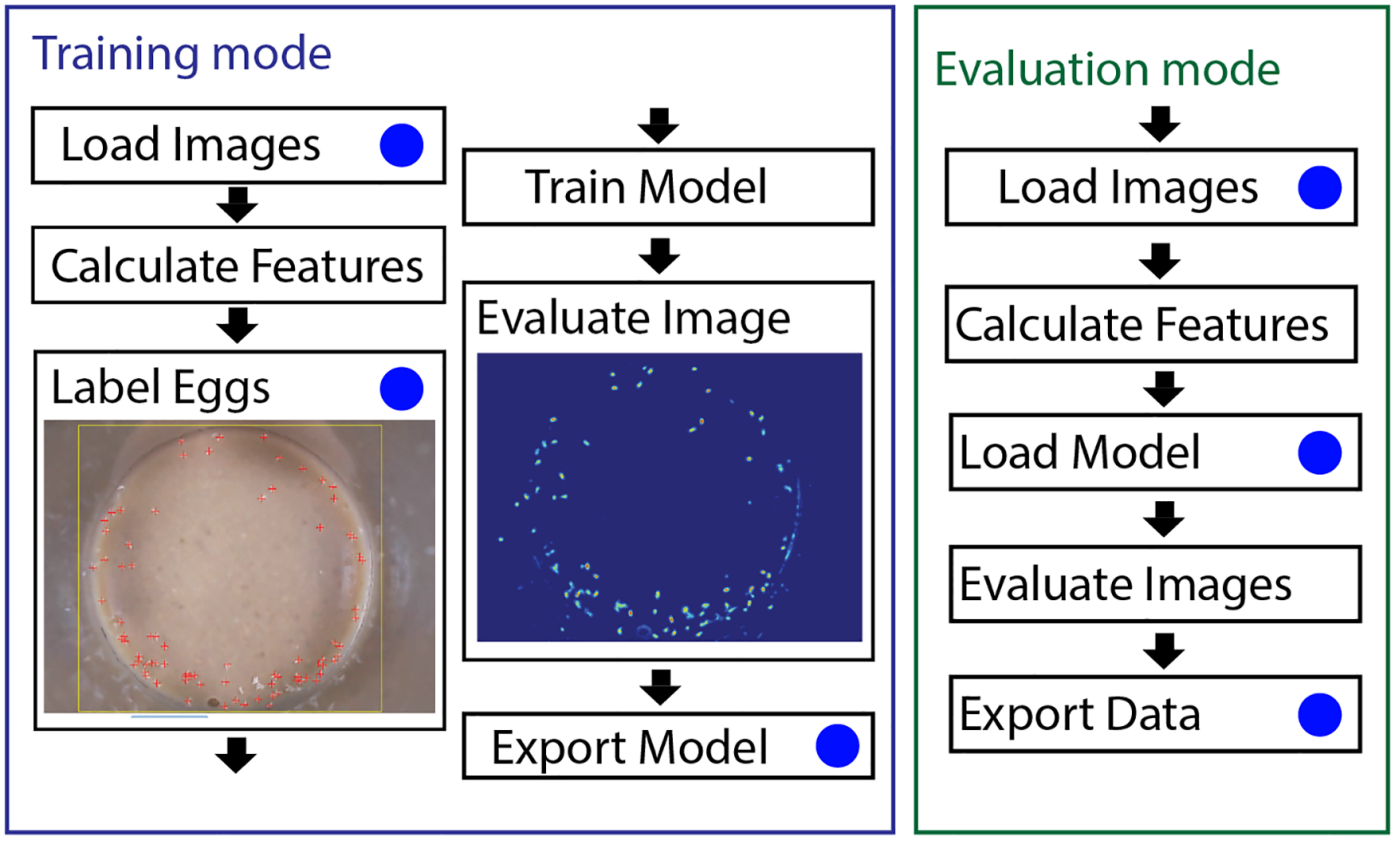
Schematic illustration of training and evaluation modes of QuantiFly software. (Left) Training mode: steps required to train a QuantiFly model to recognise eggs in an image scene. (Right) Evaluation mode: steps involved with evaluating a bulk number of images with a pre-trained model. Blue circles depict points at which a user must provide information to the system, either specifying input/output locations of files or through labelling eggs in images.

**Fig 4:**
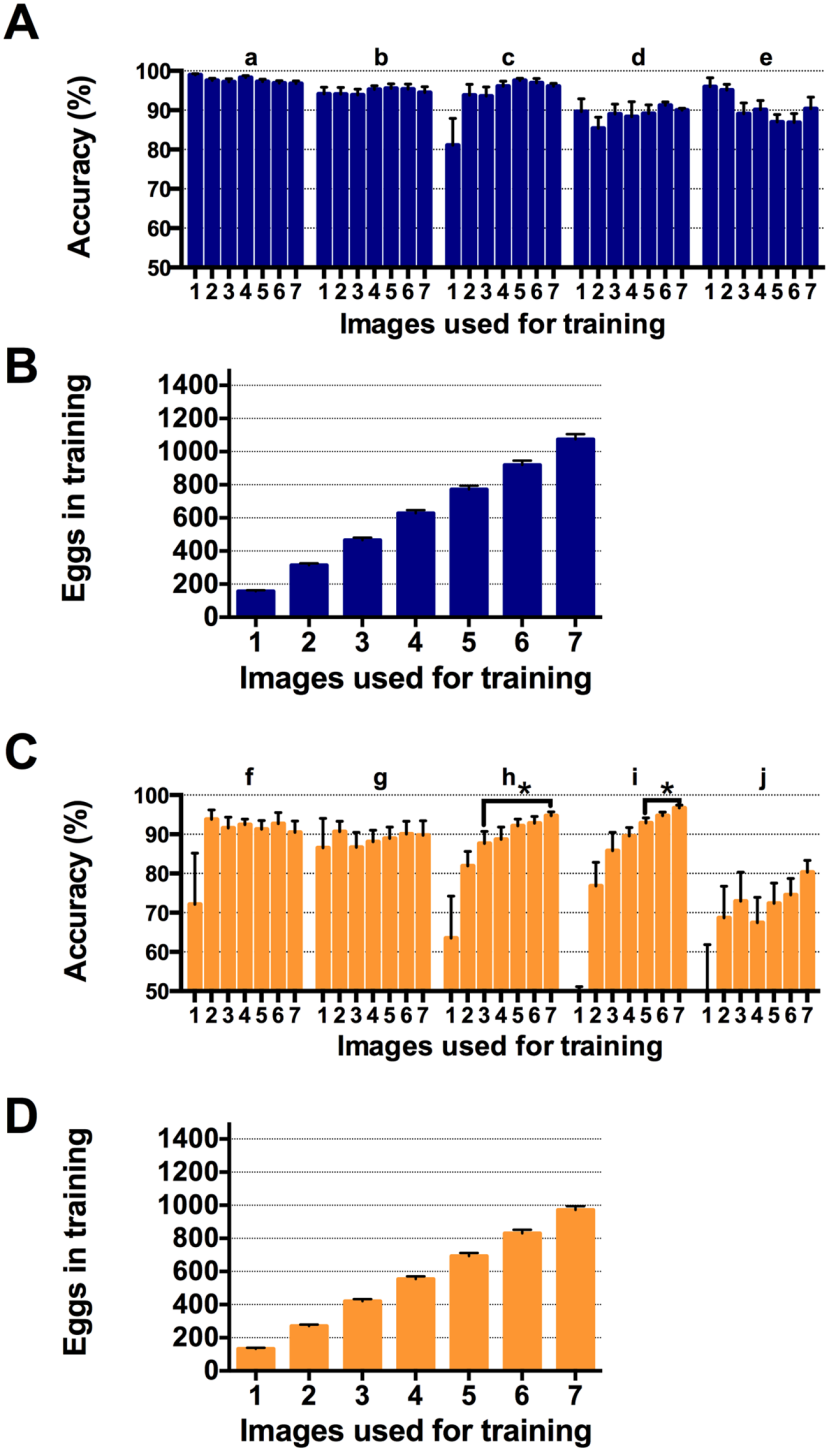
Characterisation of the quantity of training material required to achieve high prediction accuracy with QuantiFly software. The QuantiFly software accuracy was compared on transparent and opaque media datasets after training with 1–7 training images. (A) Average accuracy of algorithm when compared to digital ground-truth for transparent media datasets (a-e). (B) Average number of eggs labelled for each level of training for transparent media dataset. (C) Average accuracy of algorithm when compared to digital ground-truth for opaque media datasets (f-j) (D) Average number of eggs labelled for each level of training for opaque media dataset. Each level of training was performed on every image in the dataset and repeated 5 x. for each dataset and the accuracy averaged across all data. Error bars are SE. Students t-test paired two-way analysis was performed on data (p<0.05)

The workflow for QuantiFly training involves two stages: first the user is required to train the algorithm by identifying eggs within a few ROIs on several exemplar images, and; second, the software uses the model trained from step 1 to identify eggs in a batch of images. To initiate training, the user loads an image via a conventional file-menu interface, selects a rectangular ROI within the image to annotate and then identifies and labels instances of eggs within that region (Fig 3). Once a sufficient number of regions have been selected and labelled (see Fig 4), the algorithm is trained and the input image evaluated. The evaluated image is presented to the user as a density interpretation of the original image. After inspection of this image the user can alter the corresponding training labels, either to be more inclusive by selecting areas that are poorly represented, or more conservative by excluding points that the user does not want counted (e.g. artefacts or miss-clicks). Once three or more ROI have been labelled it is then possible to correct for bias in the image. The corrected output count is displayed along with confidence intervals (95%) which provide some feed-back as to the effectiveness of the correction. The more narrow the confidence interval is around the predicted count the more certain the user can be that the correction is having a positive impact on the given image. Once this tuning phase is complete, the refined algorithm can be used to evaluate a batch of images and the data is returned to the user as counts in a spreadsheet.

### Training Requirements of QuantiFly Software

The flexible QuantiFly training interface allows whole images or sub-regions of images to be labeled and submitted for training. For a given image or media type however, it is desirable to know how much training is required to achieve top predictive performance. To assess this, we took 8 images with corresponding ground-truths and used a cross validation strategy to characterise the accuracy. For this, one of the 8 images in a group was omitted from training and an increasing number of the remaining 7 images was used to train a different naïve model. With each of these 7 trained models, the system was used to evaluate the unseen 8th image and the accuracy of the prediction calculated by comparing it to the ground-truth count for that vial. This whole process was then repeated seven more times with the unseen test image being rotated for each iteration. Finally, the entire sequence was repeated for each of 10 different sets of 8 vials: 5 sets with transparent media (Fig 4a; groups a-e), and; 5 sets with opaque media (Fig 4A; groups f-j).

The quality of the QuantiFly software prediction varied depending on the number of images used in the training. In most cases the performance of the algorithm increased dramatically when the training was increased from one image to two images while a few of the datasets required only one dataset for high quality predictions (Fig 4A groups a, b, e). Training on more than two images produced significant improvements in two of the 10 groups of vials tested (Fig 4C, groups h and j)(p<0.05, Students t-test). To assure high accuracy for all types of media, we recommend 5 or more images to be used for training, which for the vials in this example, corresponded to approximately 600 labelled eggs (Fig 4B and 4D). Although QuantiFly can provide highly accurate output predictions from a modest amount of training data it is important to highlight that experiments should be designed with biological variation in mind. To this end, it is important, for a given condition, that at least 10 vials be used and potentially more depending on the magnitude of the differences expected between the conditions being studied. This level of replication should account for outliers and variation originating from biological noise and vial preparation differences.

### Performance Compared to Human Counters

**Fig 5:**
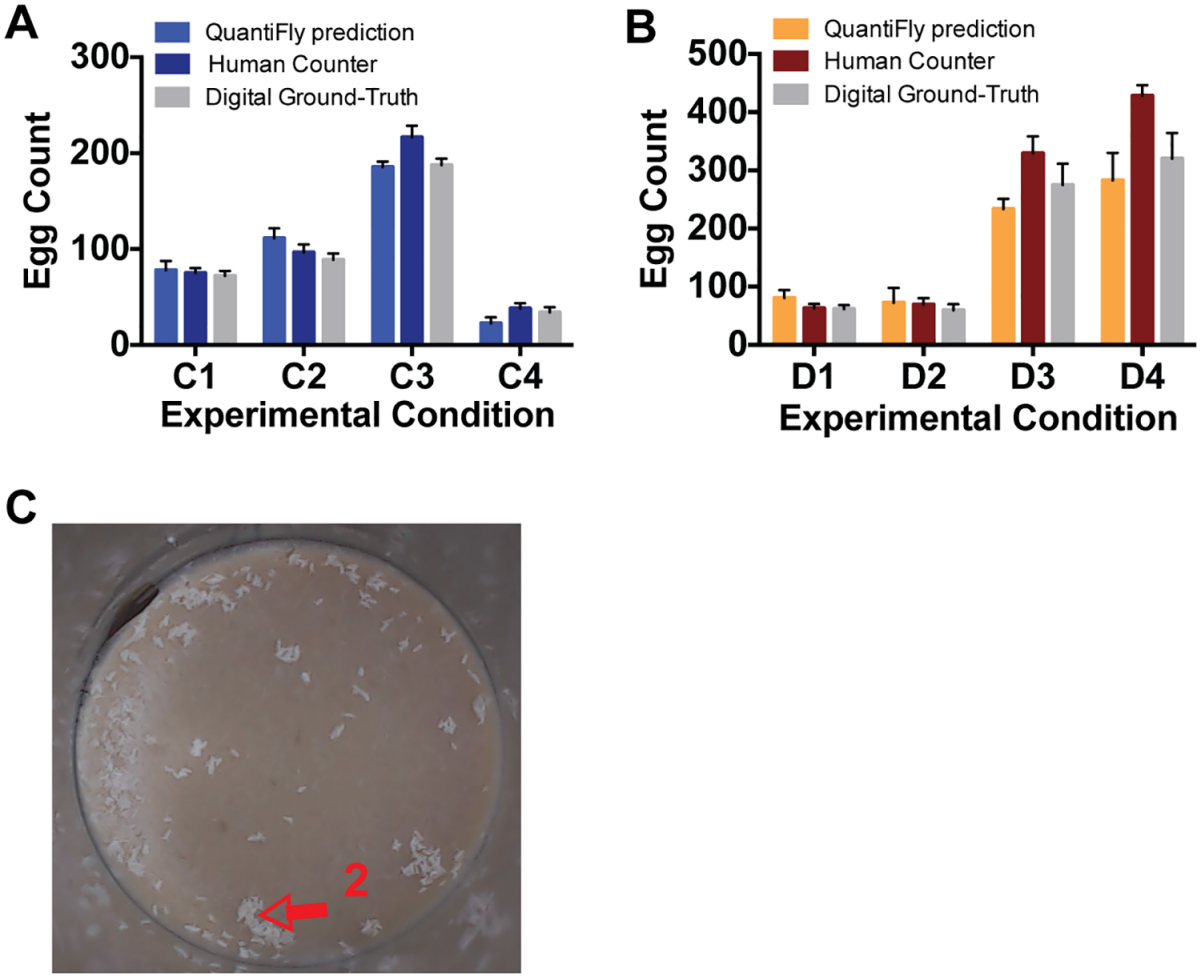
Comparison of QuantiFly performance when compared to human counter. Digital images were captured for four nutritionally different transparent media (A; C1-C4) and 4 different opaque media (B; D1-4). Estimates of the eggs in each vial were compared for the following methods: automated counts from QuantiFly algorithm; manual counts from a human and a digital on-screen ground-truth count (grey). (C) Image of opaque media vial with densely clustered eggs, red arrow 2 shows region with high-level of clustering. Error bars represent standard error of differences in vial densities in each condition (C1-4, n = 8; D1-4, n = 5 vials per condition).

The current standard for quantifying egg laying involves a laboratory researcher inspecting individual vials under a dissecting microscope and scoring eggs with a hand-held tally counter. To compare the performance of QuantiFly to that of a human, we recreated an experiment we would normally perform in our laboratory. For each of four nutritionally different translucent media, we prepared 10 vials (40 vials, Fig 5A) and for 4 different opaque media we prepared 5 vials (20 vials, Fig 5B). In each of these vials, 5 mated females were maintained for ∼18 hours, at which point the flies were removed and the eggs scored manually. The vials were then photographed under controlled lighting conditions and two randomly chosen images from each condition (8 in total) were used to train the system. In each case, a skilled human operator annotated the eggs on screen in the eight training images and used these annotations to train the system. When comparing the performance of QuantiFly versus the human manual count to assess the number of eggs per vial for all vials, we found that the data were highly similar, such that for the translucent media, all four conditions were distributed in the same way for both counting methods. Moreover, the absolute values between conditions were highly similar (Table 2). Similarly, for the opaque media, we found good concordance between QuantiFly and manual counts although at very high egg densities (200–400 eggs per vial) QuantiFly scored fewer eggs than the manual counter. (Table 2). To investigate the source of these differences, we generated a comprehensive digital on-screen count for each vial and compared these data with the manual count and QuantiFly estimates (Fig 5a and 5b). This revealed that the QuantiFly output more closely resembled the digital human counts than the manual counts (Table 2). These differences could arise due to the process of image capture obscuring some eggs that can only be counted manually, and / or, the accuracy of manual counting decreasing at very high egg densities due to egg clustering (e.g. Fig 5c) and counter fatigue.

**Table 2:** Performance of QuantiFly on transparent and opaque media compared to human manual counts and digital ground-truth counts for each dataset.

Media:	Defined Media (transparent)									
Condition: C1		Pairwise			Fold Difference			correlation		
	(count±SE; 10 vials)	Q	M	D	Q	M	D	Q	M	D
Q	78.1 ± 9.5		ns	ns	1.00	0.96	0.92		0.05	0.78
M	75.2 ± 5.0	ns		ns	1.04	1.00	0.96	0.05		0.42
D	72.1 ± 5.1	ns	ns		1.08	1.04	1.00	0.78	0.42	
Condition: C2		Pairwise			Fold Difference			correlation		
	(count±SE; 10 vials)	Q	M	D	Q	M	D	Q	M	D
Q	111.3 ± 10.4		ns	ns	1.00	0.87	0.80		0.89	0.88
M	96.6 ± 8.4	ns		ns	1.15	1.00	0.92	0.89		0.83
D	88.8 ± 6.7	ns	ns		1.25	1.09	1.00	0.88	0.83	
Condition: C3		Pairwise			Fold Difference			correlation		
	(count±SE; 10 vials)	Q	M	D	Q	M	D	Q	M	D
Q	185.5 ± 5.8		0.05	ns	1.00	1.17	1.01		0.57	0.65
M	216.6 ± 11.9	0.05		ns	0.86	1.00	0.87	0.57		0.90
D	187.7 ± 6.8	ns	ns		0.99	1.15	1.00	0.65	0.90	
Condition: C4		Pairwise			Fold Difference			correlation		
	(count±SE; 10 vials)	Q	M	D	Q	M	D	Q	M	D
Q	22.6 ± 6.2		ns	ns	1.00	1.69	1.51		0.95	0.96
M	38.1 ± 5.6	ns		ns	0.59	1.00	0.90	0.95		0.97
D	34.2 ± 5.2	ns	ns		0.66	1.11	1.00	0.96	0.97	
Media:	SY Media (opaque)									
Condition: D1		Pairwise			Fold Difference			correlation		
	(count±SE; 5 vials)	Q	M	D	Q	M	D	Q	M	D
Q	80.6 ± 13.5		ns	ns	1.00	0.78	0.76		0.98	1.00
M	62.8 ± 7.6	ns		ns	1.28	1.00	0.98	0.98		0.98
D	61.6 ± 6.6	ns	ns		1.31	1.02	1.00	1.00	0.98	
Condition: D2		Pairwise			Fold Difference			correlation		
	(count±SE; 5 vials)	Q	M	D	Q	M	D	Q	M	D
Q	72.1 ± 25.7		ns	ns	1.00	0.96	0.82		0.97	0.97
M	69.0 ± 11.4	ns		ns	1.04	1.00	0.86	0.97		0.96
D	59.4 ± 10.4	ns	ns		1.21	1.16	1.00	0.96	0.96	
Condition: D3		Pairwise			Fold Difference			correlation		
	(count±SE; 5 vials)	Q	M	D	Q	M	D	Q	M	D
Q	233.4 ± 17.6		ns	ns	1.00	1.41	1.18		0.91	0.75
M	329.2 ± 29.2	ns		ns	0.71	1.00	0.83	0.91		0.94
D	274.6 ± 36.9	ns	ns		0.85	1.20	1.00	0.75	0.94	
Condition: D4		Pairwise			Fold Difference			correlation		
	(count±SE; 5 vials)	Q	M	D	Q	M	D	Q	M	D
Q	282.8 ± 47.2		ns	ns	1.00	1.51	1.13		0.67	0.56
M	428.2 ±18.4	ns		ns	0.66	1.00	0.75	0.67		0.77
D	320.2 ± 44.1	ns	ns		0.88	1.34	1.00	0.56	0.77	

Q:QuantiFly prediction, M: Manual human counts, D: Digital ground-truth counts. Pairwise: Tukeys pairwise comparison; Fold Difference; the fold difference between counts; Correlation, Pearsons correlation coefficient for each comparison.

### Sample Size Calculation and Time Analysis

A major advantage to an automated system for egg counting is its ability to save time. For the transparent media shown in Fig 5A, the human manual counts took on average 48.9 ± 3.9 s for each vial, taking 33 min in total for all 40 vials. In contrast, for the QuantiFly software, 10 min was required to annotate images and to train the software and only a few seconds was then required for the software to evaluate each image in the dataset. Thus, we achieved a significant time saving with only a relatively small experiment and since the time investment into training is only made once, the user would save an additional ∼45 seconds for each additional vial added to the experiment.

**Fig 6:**
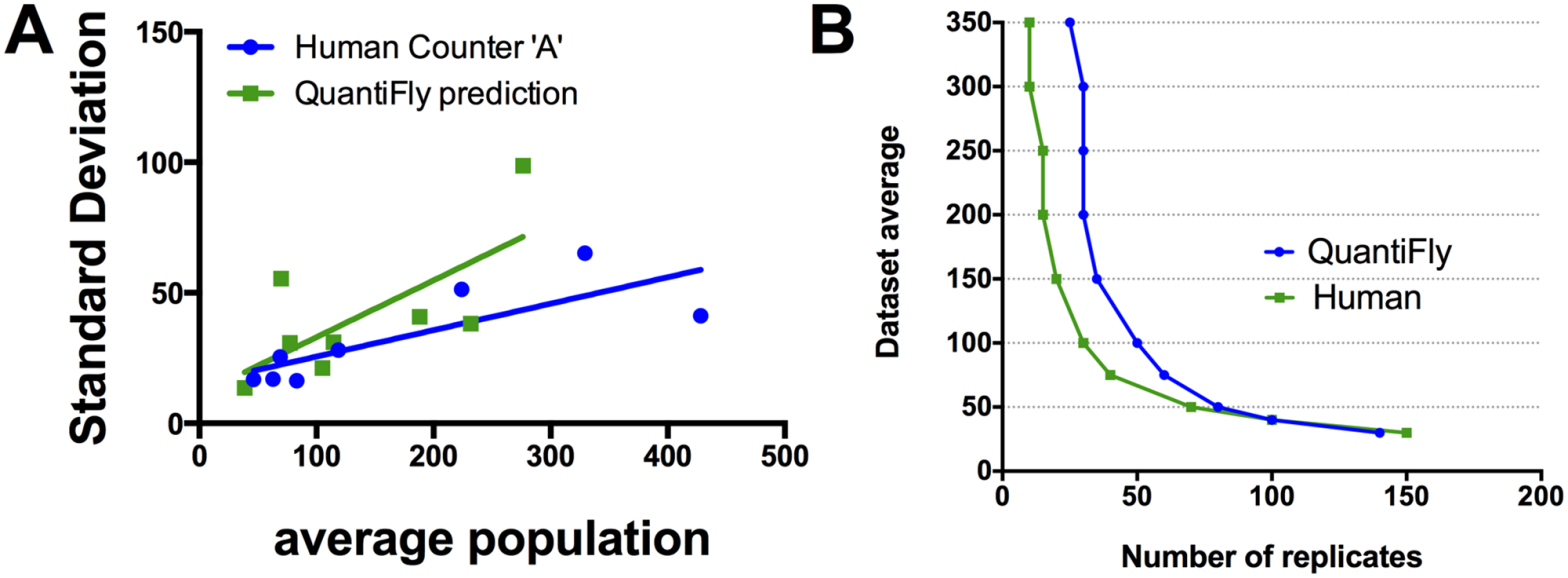
Sample size calculation. (A) The number of replicates required to achieve a confidence interval of below 0.05 was calculated for the manual human count compared to the QuantiFly software using population standard deviation calculated from Fig 5A and 5C. Plot represents the number of replicates required to separate conditions which differ by 1.1 fold. (B) Projected time requirements for counting vials for a single condition using the existing manual approach or the QuantiFly software.

To illustrate this saving, we found the relationship between the average vial count and the standard deviation for each condition in Fig 5 and calculated the number of replicates required to achieve significance for a 1.1 fold egg count difference (Fig 6A). For a researcher to distinguish conditions, which for example are 50 and 45, would take 70 replicates for manual counts and 80 replicates for QuantiFly. This would take a human counter approximately one hour of consistent counting whereas this experiment would take approximately 13 minutes using QuantiFly (Fig 6B). In fact, we predict QuantiFly should save time for any single food type experiment in which more than 13 vials need to be counted (Fig 6B). Furthermore, given that the main overhead with the QuantiFly software is the training phase, we have made it possible to evaluate the performance of more than one pre-saved model on the given data without significant investment of time. If a batch of models had been saved from previous training data then it is straight-forward with the software to evaluate more than one model and to compare the results. If acquisition settings are particularly consistent over-time then it may be possible to reuse models from previous experiments without the need to retrain each time, thus saving additional time.

### Conclusions

We report the testing, optimisation and implementation of a machine-learning technique for the task of automating *Drosophila* egg counting. In doing so, we transformed a conceptual algorithm accessible only to computer scientists, into a user-friendly piece of software called QuantiFly. During this process we identified a limitation in the baseline algorithm and corrected for it with a simple transformation improving the accuracy of the algorithm. The software is available and compiled for the three major operating systems and the only additional equipment required is to provide a means to capture digital images of the eggs. For this purpose, we have also designed a box that serves as a camera mount and offers controlled lighting conditions. For plans and pictures of this hardware, as well as the possibility of ordering it, please visit:http://polygonaltree.github.io/egg_counter/. QuantiFly is both accurate and offers enormous timesaving potential to any individual facing the unenviable task of counting fly eggs.

## Supporting Information

S1 Fig Vial Images used in study. Images of vials used in datasets a-j and in conditions C1-C4 and D1-D4 for human manual count versus QuantiFly predictions.
(PDF)

S1 Text User manual for using QuantiFly software. Step-by-step instructions for the training of QuantiFly software and also how to perform bulk evaluation of images.
(PDF)
