# Supplementary material for: QuantiFly: Robust Trainable Software for Automated Drosophila Egg Counting
Source: PLoS One. 2015 May 18;10(5):e0127659. doi: 10.1371/journal.pone.0127659 (PMC4436334; doi:10.1371/journal.pone.0127659)
Supplement: S1 Fig — Images of vials used in datasets a-j and in conditions C1-C4 and D1-D4 for human manual count versus QuantiFly predictions. (PDF) [file pone.0127659.s001.pdf]

# Supplementary Figure

Dominic Waithe

April 15, 2015

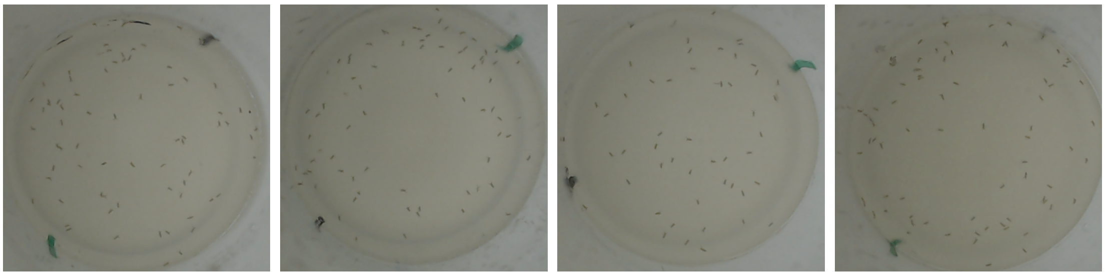

(a) image001 (b) image002 (c) image003 (d) image004

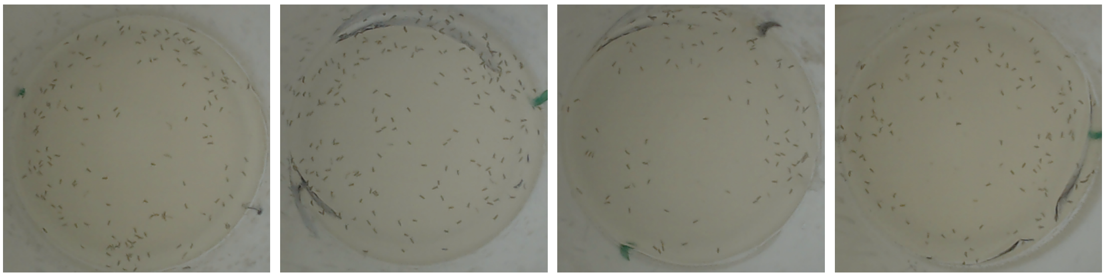

(e) image005 (f) image006 (g) image007 (h) image008

Figure 1: Dataset A

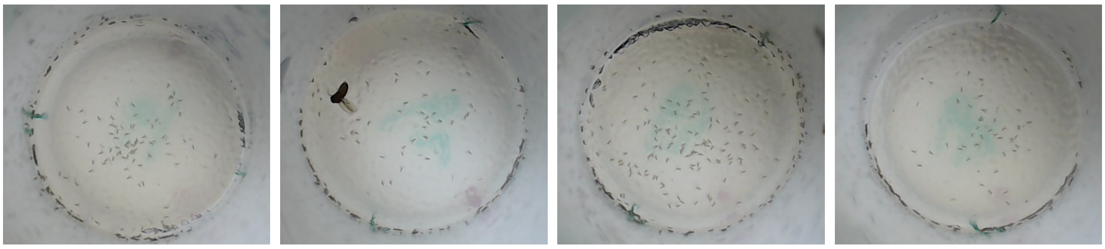

(a) image001 (b) image002 (c) image003 (d) image004

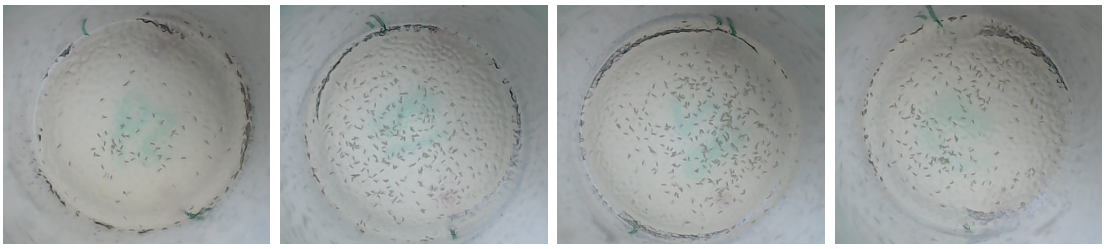

(e) image005 (f) image006 (g) image007 (h) image008

Figure 2: Dataset B

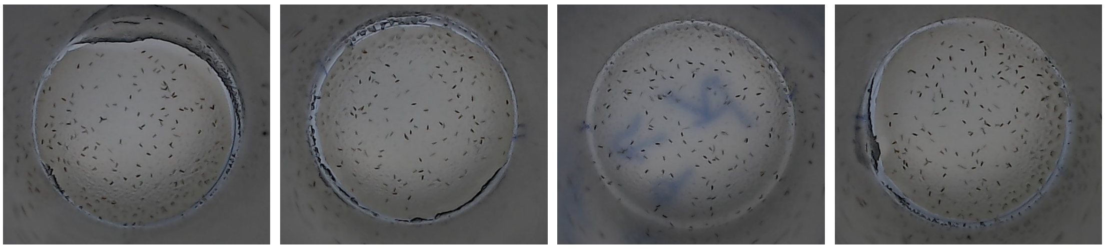

(a) image001 (b) image002 (c) image003 (d) image004

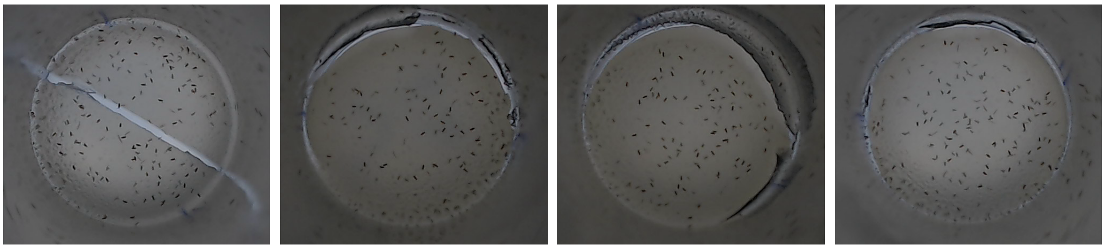

(e) image005 (f) image006 (g) image007 (h) image008

Figure 3: Dataset C

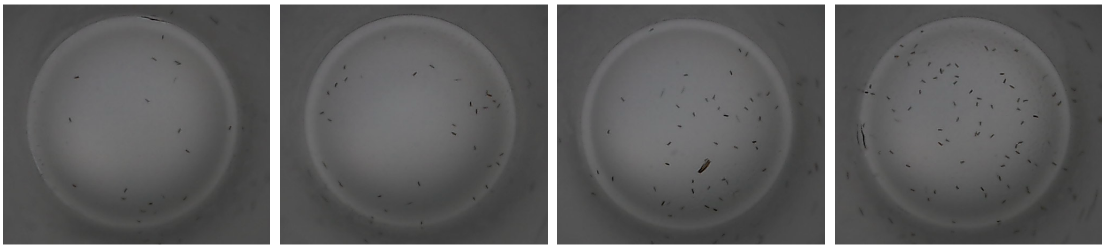

(a) image001 (b) image002 (c) image003 (d) image004

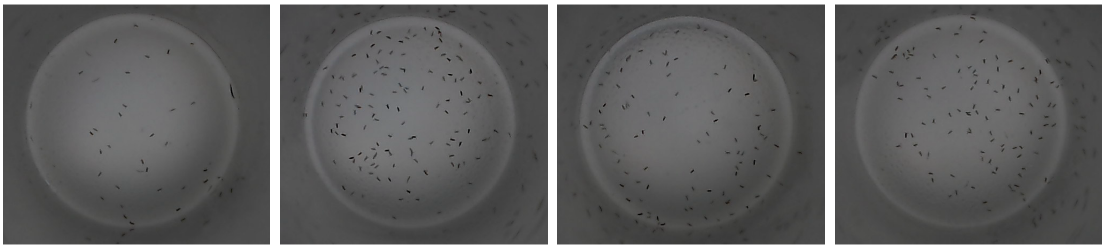

(e) image005 (f) image006 (g) image007 (h) image008

Figure 4: Dataset D

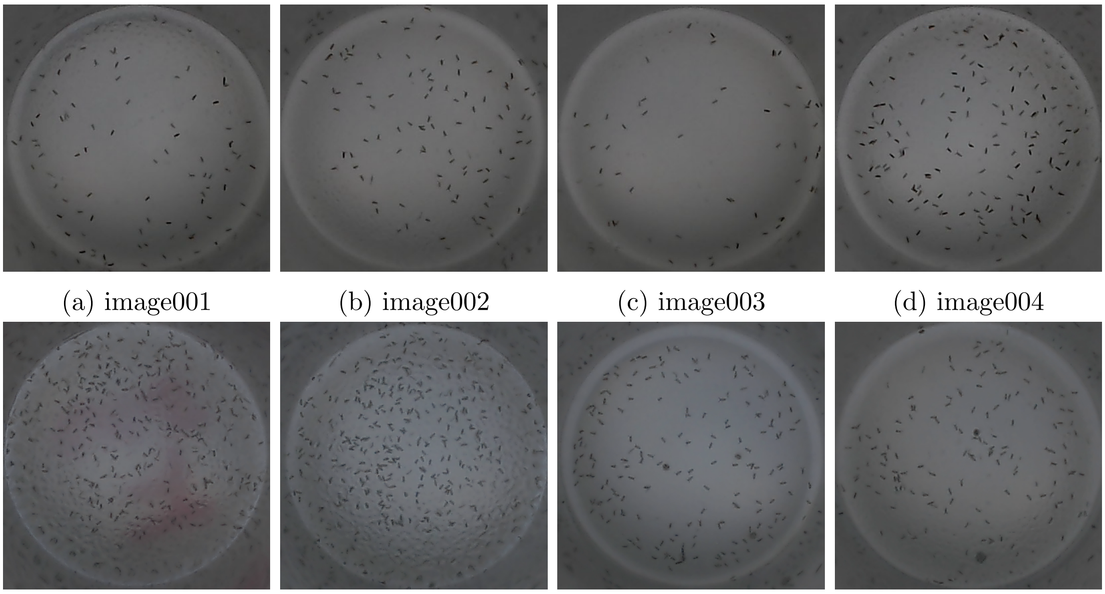

(a) image001 (b) image002 (c) image003 (d) image004

(e) image005 (f) image006 (g) image007 (h) image008

Figure 5: Dataset E

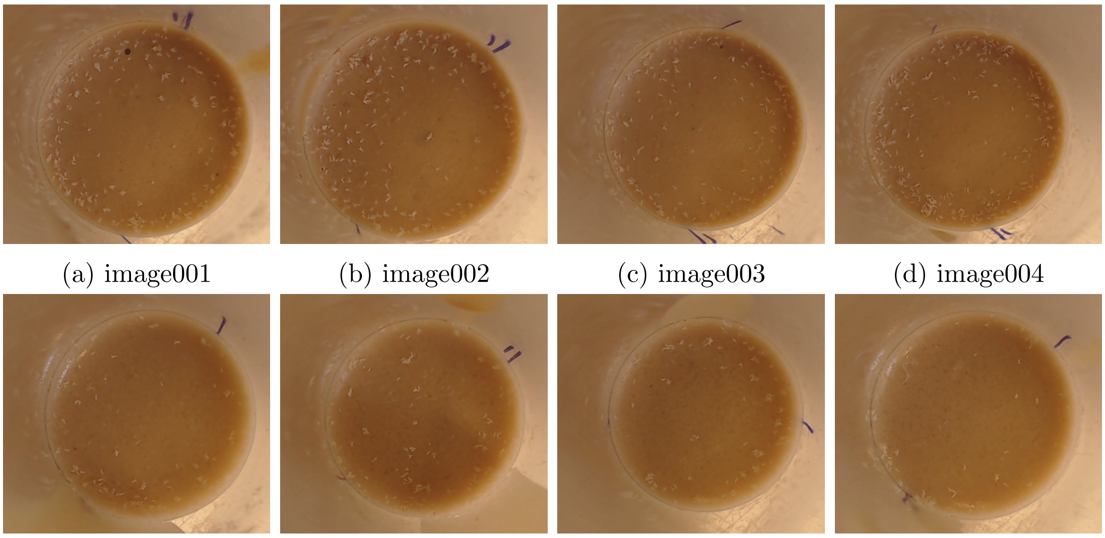

(a) image001 (b) image002 (c) image003 (d) image004

(e) image005 (f) image006 (g) image007 (h) image008

Figure 6: Dataset F

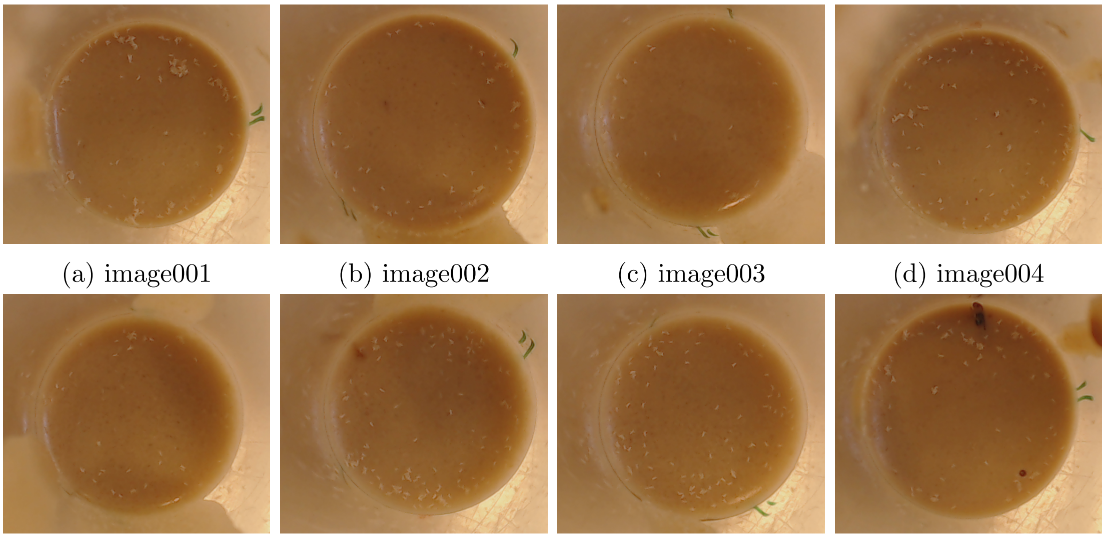

(a) image001 (b) image002 (c) image003 (d) image004

(e) image005 (f) image006 (g) image007 (h) image008

Figure 7: Dataset G

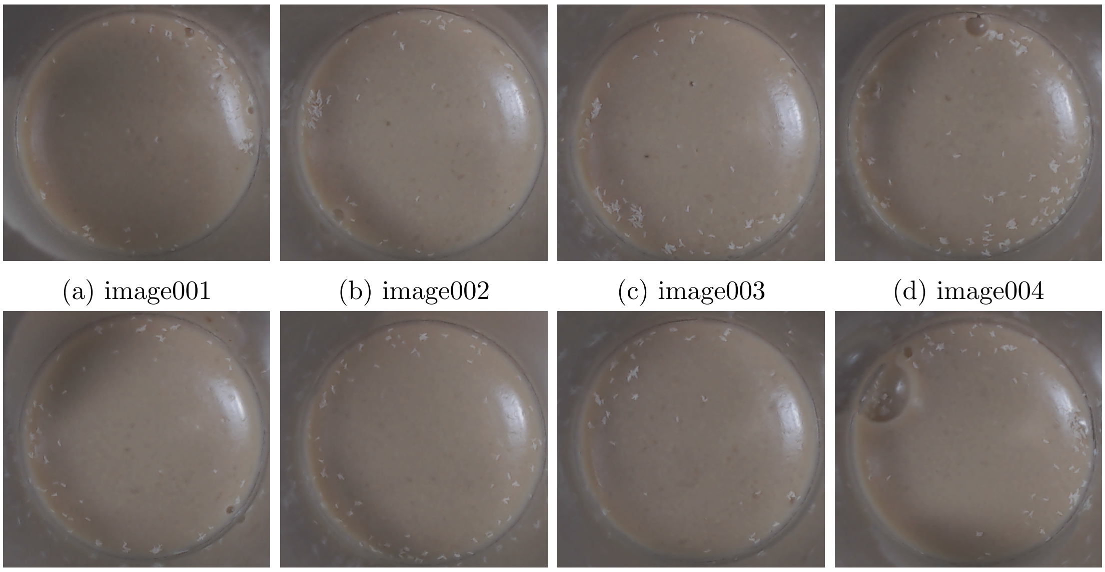

(a) image001 (b) image002 (c) image003 (d) image004

(e) image005 (f) image006 (g) image007 (h) image008

Figure 8: Dataset H

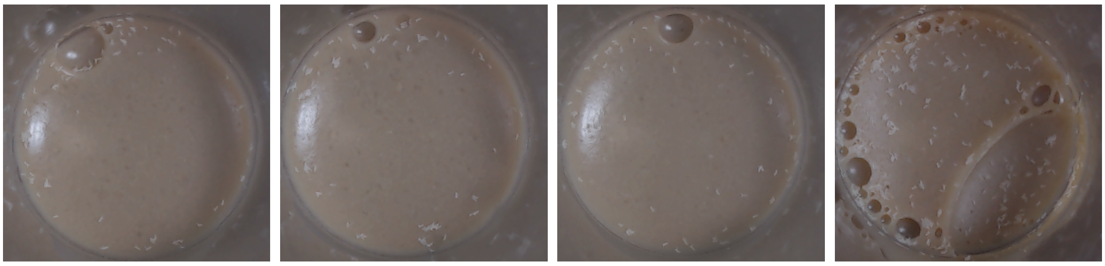

(a) image001 (b) image002 (c) image003 (d) image004

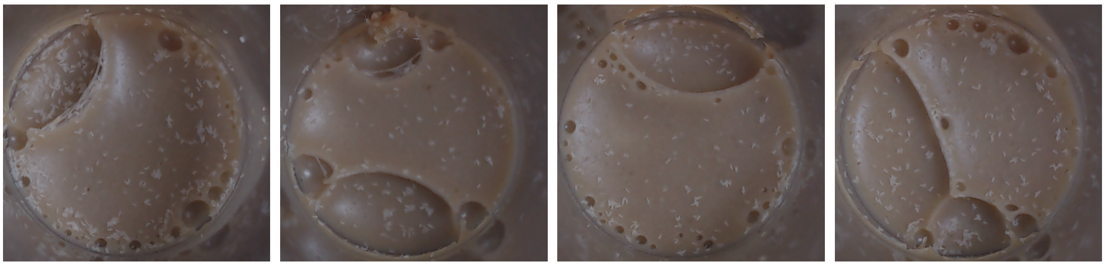

(e) image005 (f) image006 (g) image007 (h) image008

Figure 9: Dataset I

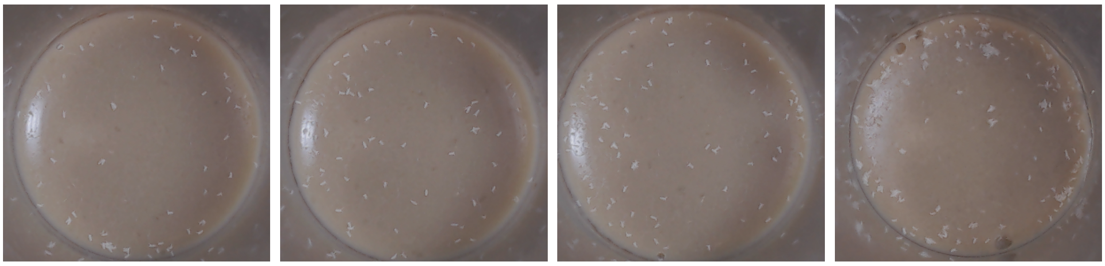

(a) image001 (b) image002 (c) image003 (d) image004

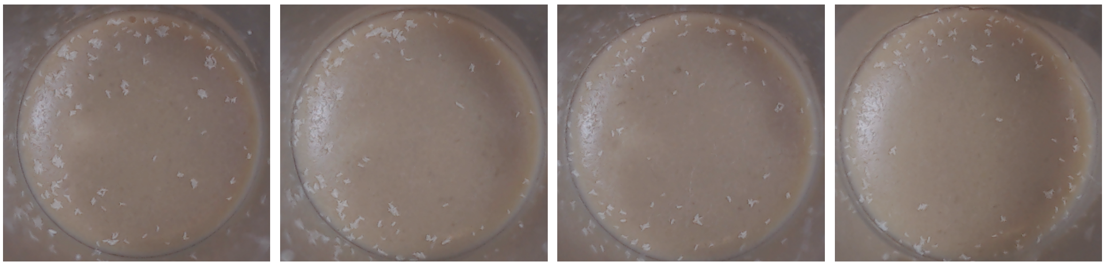

(e) image005 (f) image006 (g) image007 (h) image008

Figure 10: Dataset J

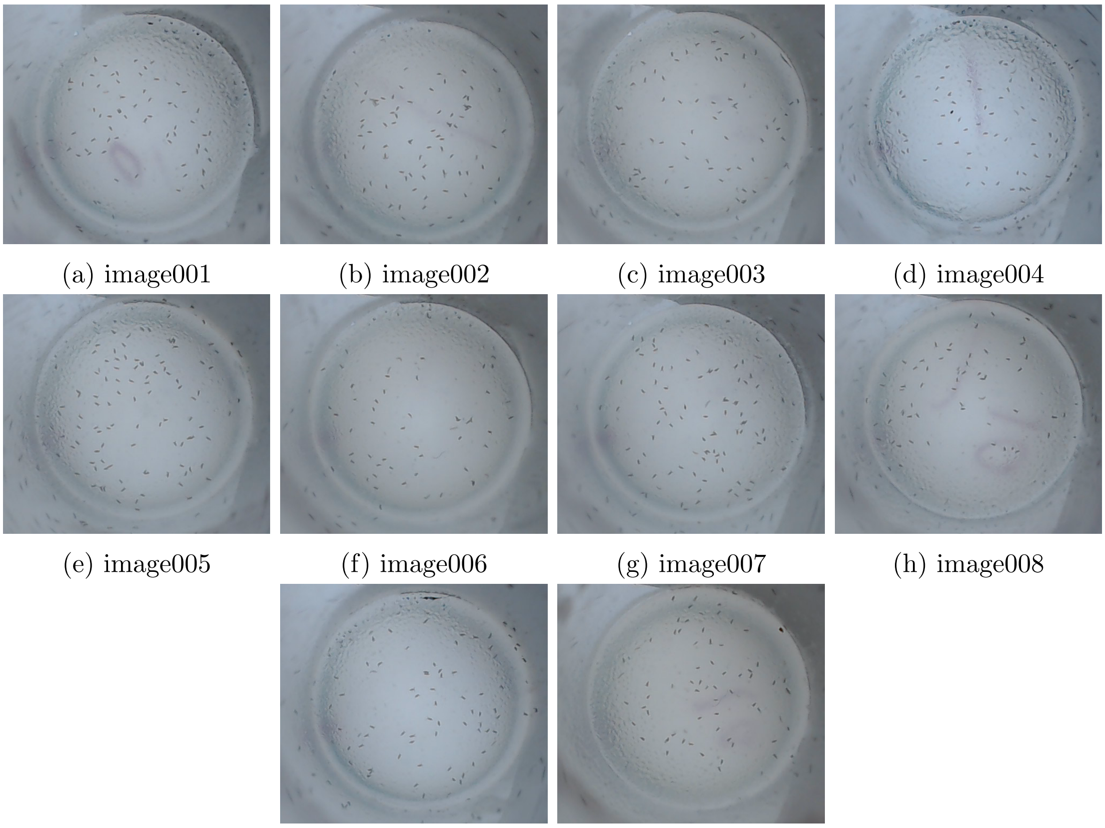

(a) image001 (b) image002 (c) image003 (d) image004

(e) image005 (f) image006 (g) image007 (h) image008

(i) image009 (j) image010

Figure 11: Dataset C1

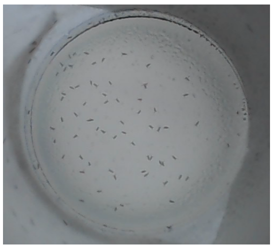

(a) image001

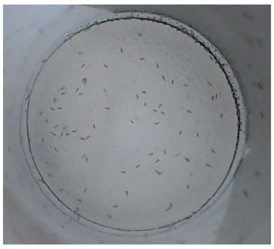

(b) image002

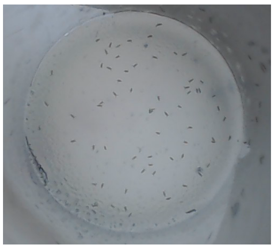

(c) image003

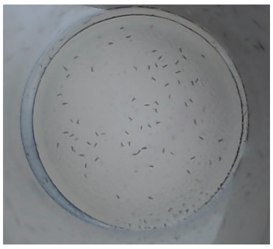

(d) image004

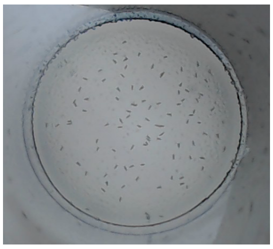

(e) image005

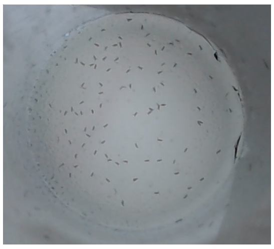

(f) image006

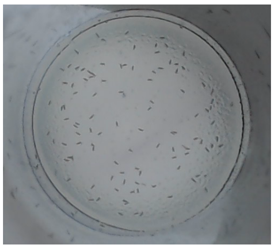

(g) image007

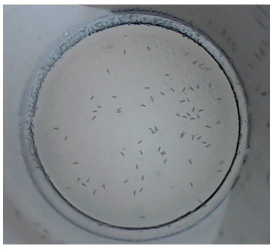

(h) image008

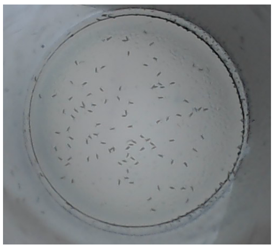

(i) image009

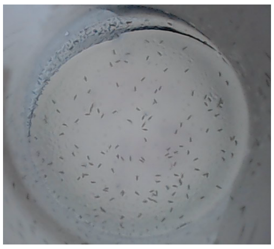

(j) image010

Figure 12: Dataset C2

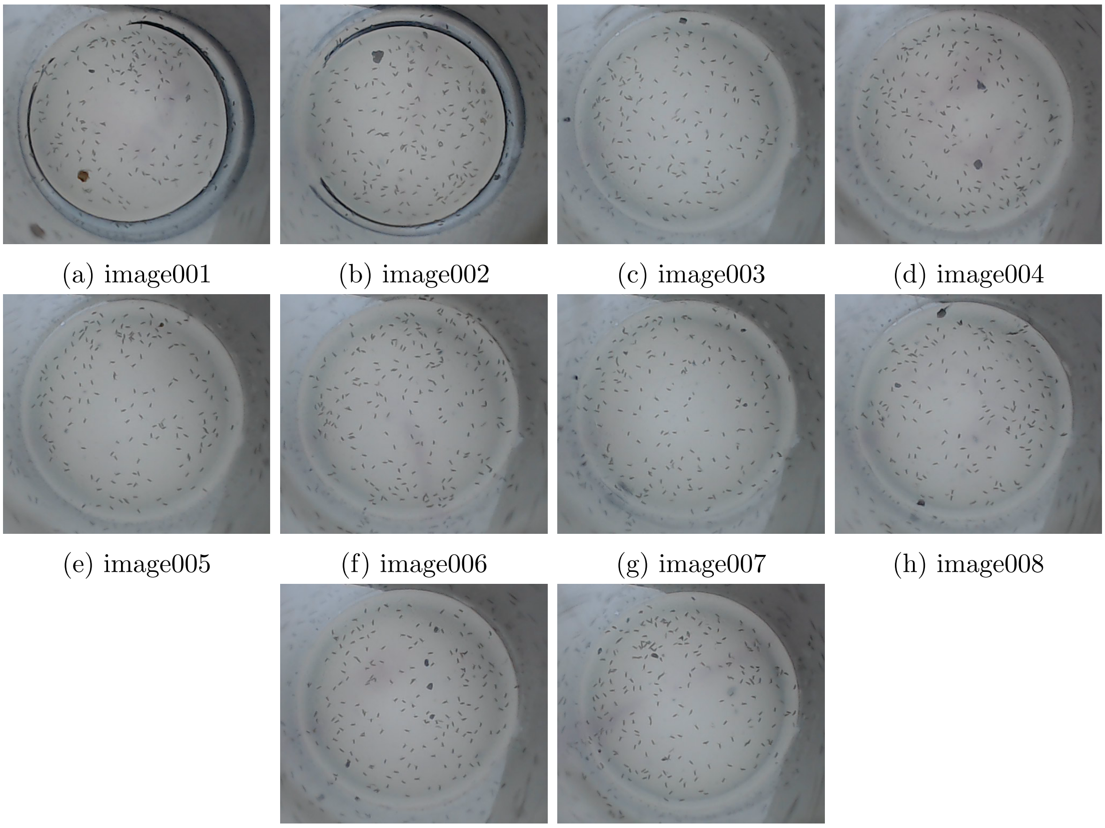

(a) image001 (b) image002 (c) image003 (d) image004

(e) image005 (f) image006 (g) image007 (h) image008

(i) image009 (j) image010

Figure 13: Dataset C3

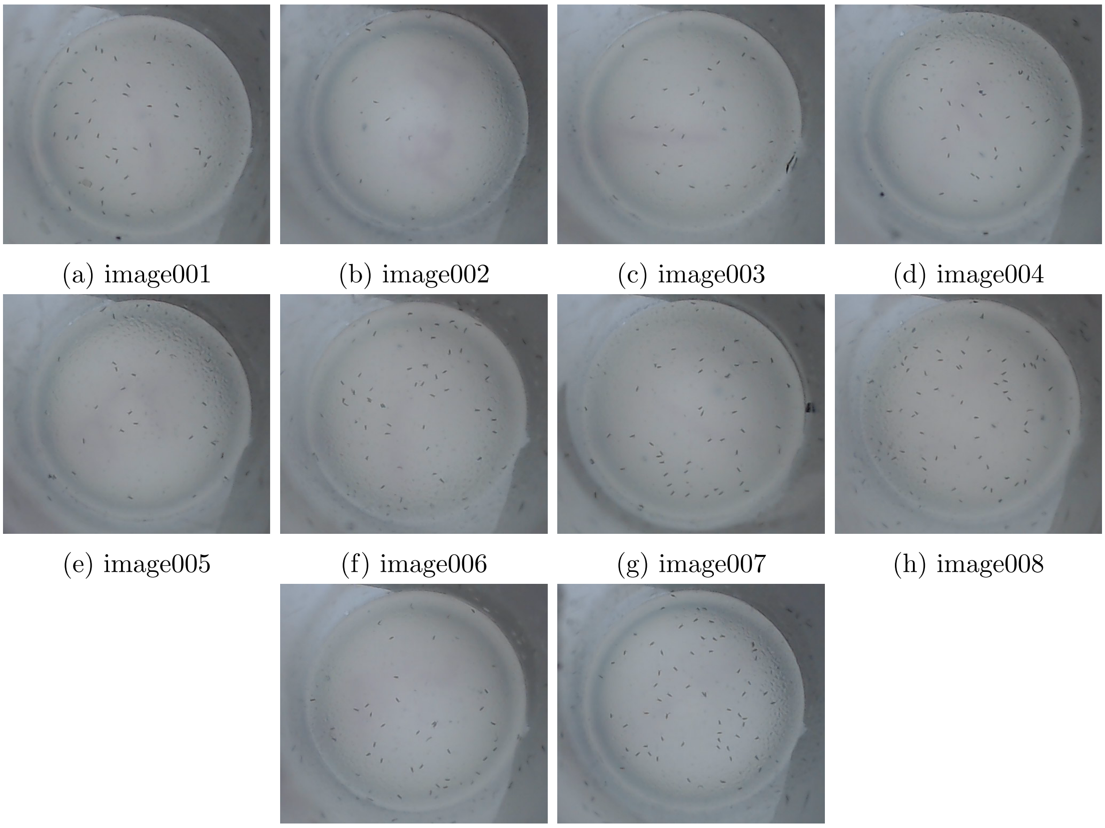

(a) image001 (b) image002 (c) image003 (d) image004

(e) image005 (f) image006 (g) image007 (h) image008

(i) image009 (j) image010

Figure 14: Dataset C4

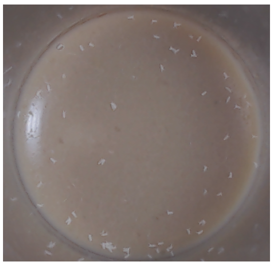
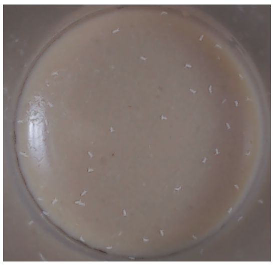
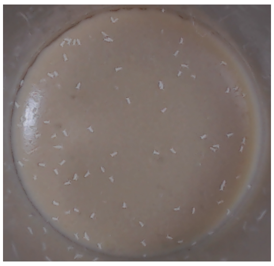
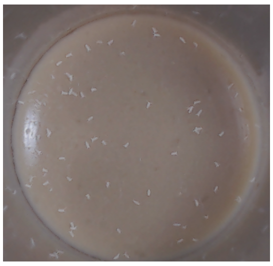

(a) image001 (b) image002 (c) image003 (d) image004

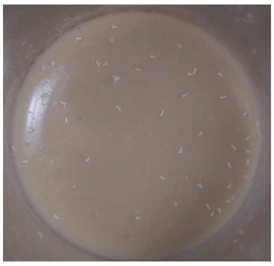

(e) image005

Figure 15: Dataset D1

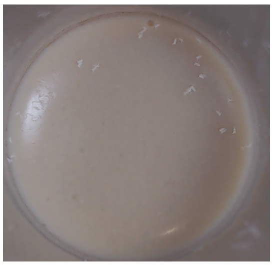
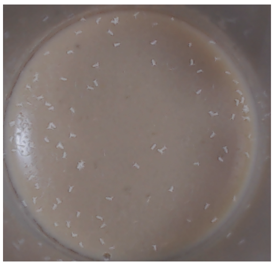
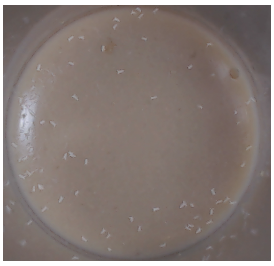
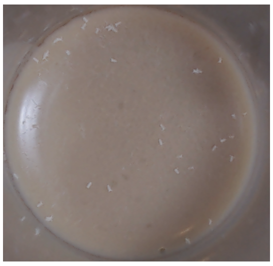

(a) image001 (b) image002 (c) image003 (d) image004

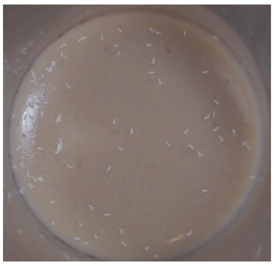

(e) image005

Figure 16: Dataset D2

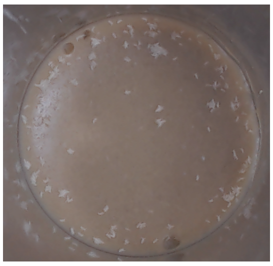
(a) image001

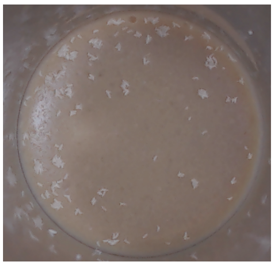
(b) image002

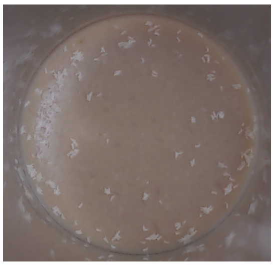
(c) image003

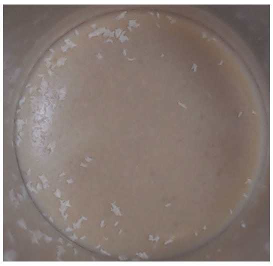
(d) image004

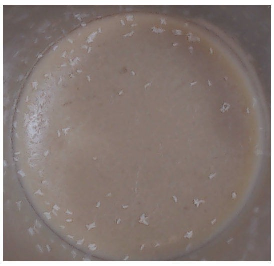
(e) image005

Figure 17: Dataset D3

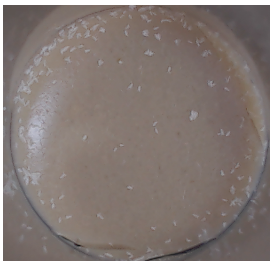
(a) image001

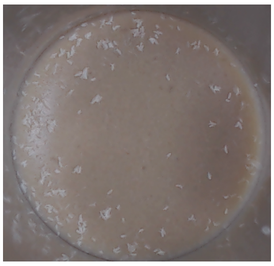
(b) image002

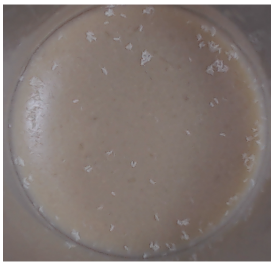
(c) image003

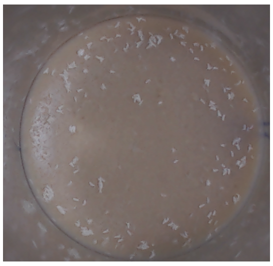
(d) image004

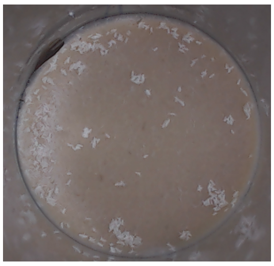
(e) image005

Figure 18: Dataset D4
