# Supplementary material for: QuantiFly: Robust Trainable Software for Automated Drosophila Egg Counting
Source: PLoS One. 2015 May 18;10(5):e0127659. doi: 10.1371/journal.pone.0127659 (PMC4436334; doi:10.1371/journal.pone.0127659)
Supplement: S1 Text — Step-by-step instructions for the training of QuantiFly software and also how to perform bulk evaluation of images. (PDF) [file pone.0127659.s002.pdf]

QuantiFly Software Guidelines

By Dominic Waithe 2015.

Introduction

The QuantiFly software is an implementation and optimisation of the density estimation technique first conceived of by Lempitsky and Zisserman [1] and than later refined by Fiaschi et al. [2]. This software provides a gui for the training and optimisation of the density kernel method of evaluating the number of object instances in an image. The interface allows fast and informative training to be performed with labelling of user-selected regions. The software allows the processing of single and multi-channel images and also provides a variety of feature descriptors of varying complexity. The software is also optimised for evaluation of bulk number of images. The following document is a user-guide for training and utilising the software.

File preparation:

Image files come in many shapes and sizes. To make a program which can handle all types of image is a very demanding task. The QuantiFly software is compatible with both 'PNG' and 'TIFF' formats. Hyper-stack TIFFs are not currently supported and so it is necessary to convert image stacks into RGB format before using the QuantiFly. This means that presently up to only three colour channels can be supported.

Running QuantiFly training software:

1) Locate the 'QuantiFly-training' software which you should have downloaded and unzipped from the website https://github.com/dwaithe/quantifly

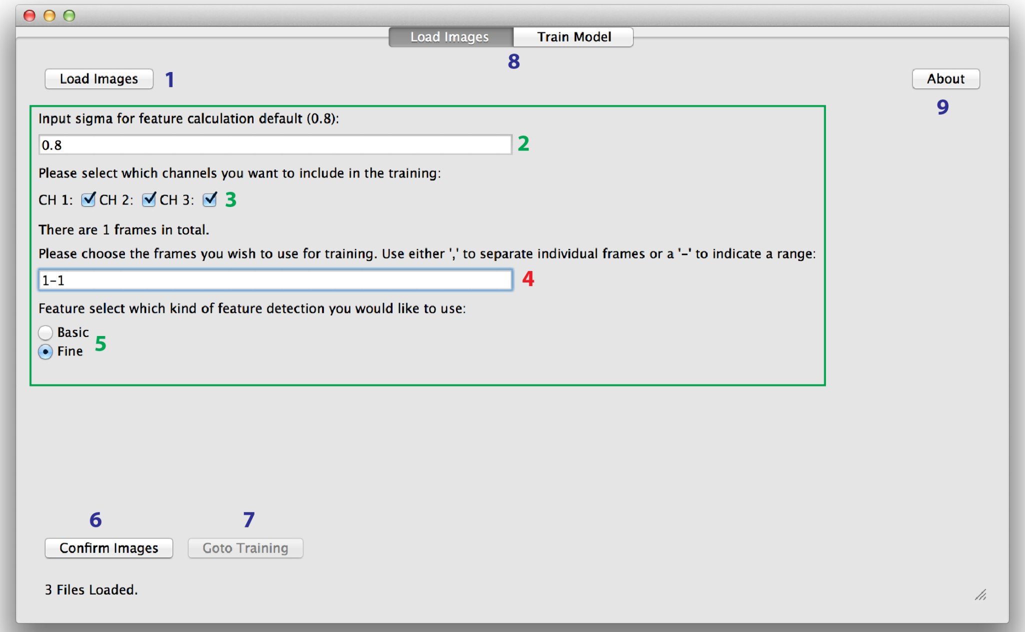

2) When the program first loads you will be presented with a button which reads 'Load Images' (1). Clicking this will bring up a dialog box where you can select one or more files to load. Presently, files must be either in the format 'TIFF' or 'PNG' format (see file preparation above for further details). It is possible to load one or more file in the same folder by selecting multiple using either the 'Ctrl' or 'Shift' key. Click the 'Open' button to commit the images.
3) Once the 'Open' button is pressed the images are inspected and further dialog options appear (green box). The input sigma for the feature selection represents the scale of the feature calculation (2). The default is 0.8. Leave this value if you are unsure of its meaning. The smaller the value is, the more likely the model will detect fine-grain features. The larger it is, the less sensitive the detection is to smaller details in comparison to larger features.
4) The channel tool (3) is used to determine which combination of colour channels the algorithm is applied on. Important: if you only want the model to consider the information in a particular channel and not another channel, you must select accordingly here. The model will only see data present in the channels which are selected here. Due to the increased information the model takes longer to train when multiple channels are selected.
5) If you image is a TIFF image with multiple slices then the frames dialog will appear (4). For training of images it is often not necessary to include all the images present in an image stack or an image time-series. This dialog allows you to select individual images or ranges of images from the available slices. The number of frames in the image stack will be shown above the dialog. To select images, use either a ',' to select individual images or a '-' to indicate a range. E.g 10-20 would include image slices 10 through 20 in the processing, where as 10, 20 would include frame 10 and

20 only. It is also possible to use the less than '<' and greater than '>' operator to define a range. The more frames included in training the more processing is required and so the slower the algorithm.

6) The final parameter to be selected is the type of features to be calculated. All the features are related and represent different levels of complexity (5). 'Fine' is the default, and is better than 'Basic' but takes longer to calculate.
7) Once the parameters have been set use the 'Confirm Images' button to upload the desired images and calculate the image features (6). This may take some time. Progress can be established by reading the dialog just below this button.
8) When all the images have been loaded and the features calculated the 'Goto Training' button will become clickable (7). Click this button to proceed to training. At any point alternate settings can be set by returning to this area of the program by clicking the 'Load Images' tab at the top of the screen (8). Just remember to click the 'Confirm Images' button to reprocess the images with the desired settings.

Model Training:

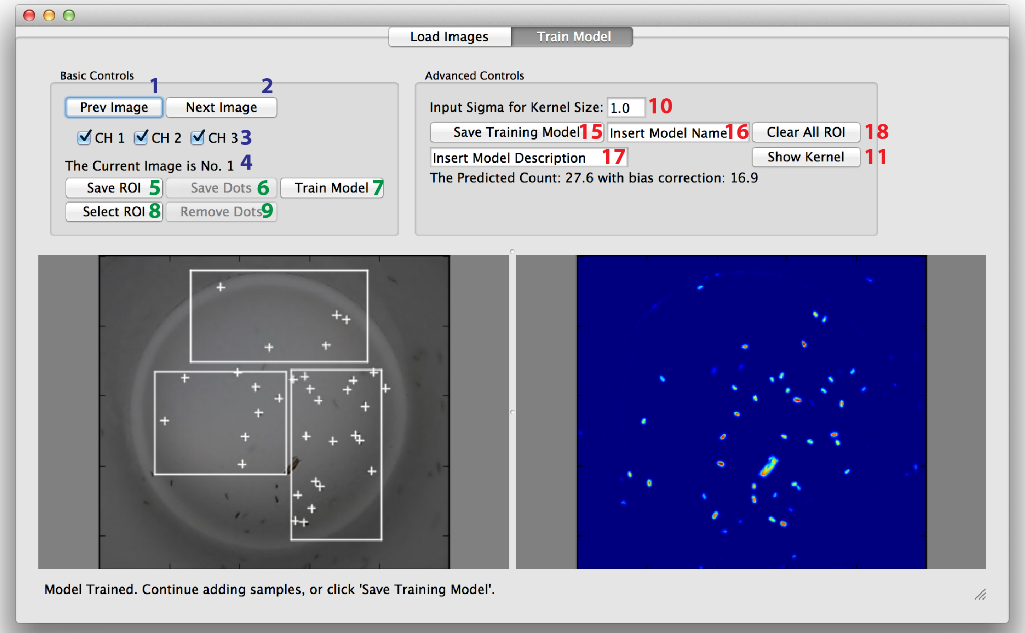

Once the 'Goto Training' button has been clicked the following application tab will appear entitled 'Train Model'. In this window of the Quantifly application it is possible to navigate through your images or stack slices and also to train the system to recognize objects within your images. The above figure represents the graphical interface used for the data visualization and model training. Items 1-4 in the above figure represent the controls for navigating through your images and selecting which channels to visualize. Buttons 5-9 represents the tools used for training the system. Whereas, inputs 10-18 are advanced tools for output of trained model and starting again.

Image navigation

Upon loading of the 'Train Model' tab your images should appear on the left of the screen. To navigate these images use either the buttons marked 'Prev Image' (1), 'Next Image' (2) or use the ',' or '.' Buttons on the keyboard, alternatively roll the mouse-wheel. The current image number is displayed towards the top-right of the screen (4). Your image may have up to 3 colour channels. Although those channels contributing data to the training are set in the previous section, it is still possible to visualize each channel independently using the channel check-boxes (3).

Instance selection and model training

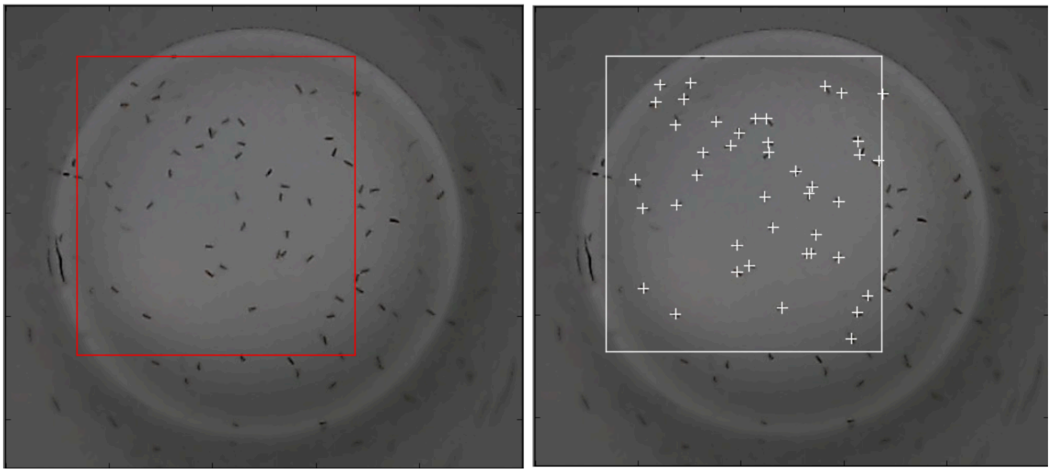

The QuantiFly interface gives the ability to quickly and selectively train a model to recognise objects of interest. This facility is performed using the tools below the image pane (5-9) and the above figure illustrates some of the main actions. To perform the training start by clicking and then dragging directly on the image pane. A region should appear which can be re-drawn any number of times by left-clicking, dragging and releasing the mouse over the loaded image. This region of interest (ROI) represents the pixels, which are going to be submitted to the training algorithm. To save the region, click the 'Save ROI' button(5). Next we have to tell the algorithm where the objects are within the ROI of interest. To do this, simply click on the centre of the object. It is important to exhaustively label all the objects which are present within your region. Once all the objects are labelled click 'Save Dots' to commit the labelled instances to that region(6). At this stage the density estimate kernel will appear on the right-hand pane of the screen. This is shown in the below figure.

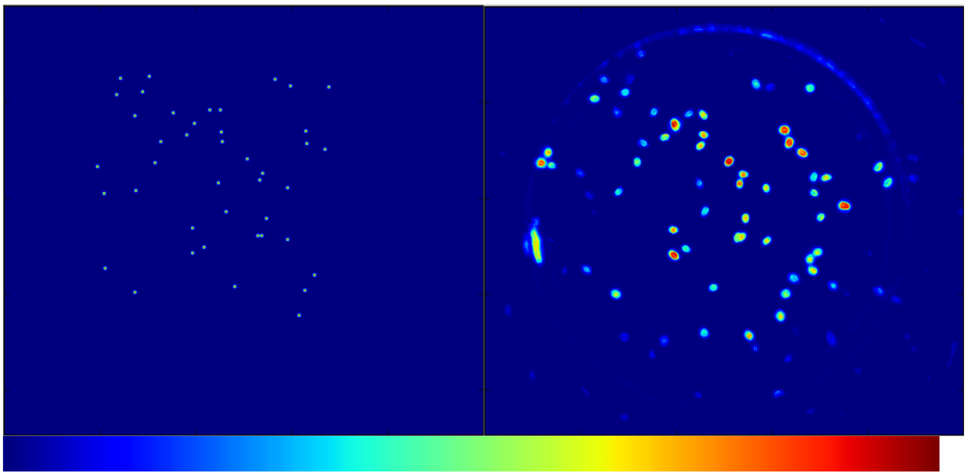

Once you have selected and labelled instances in a least one region it is possible to train the model. To train the model click the 'Train Model' button (7). After a short-time the output of the trained model will appear in the right-hand image pane replacing the density estimate kernel image. The image is pseudo-coloured using the 'jet colour scheme. Bluer tones represent lower densities whereas redder tones represent higher densities. Even though the training was performed only on a small number of object instances the model is applied globally to all pixels within each image. The more regions and instances which are labelled the more accurate the model will be. Exhaustive labelling of all instances is not required however to get accurate results. The 'Show Prediction' and 'Show Kernel' buttons allow you to switch between the above modes at any time (11).

Parameter Optimisation

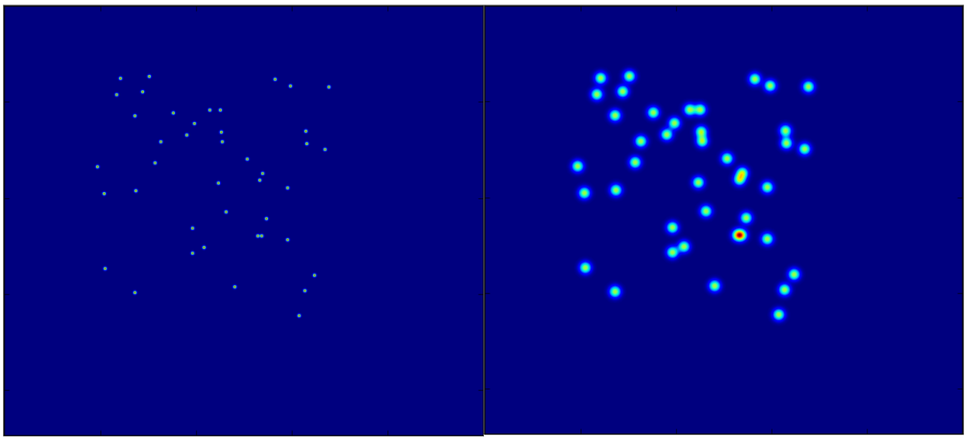

Depending on your application it may be necessary to tweak the parameters of the model to optimise the object feature description and training. The first parameter which can be tweaked is the 'Input sigma for kernel size:' parameter (10). Changing this parameter changes the size of the kernel used to model the object. Although not an exact science the kernel should represent approximately 70 % of the object size. This parameter, in theory, should not be set to be too small as it will mean only a few features are used to describe the object centre. In practise however human error means the instance labelling is not perfect and so

the centre of object contains some variation. Therefore good results are often achieved using a range of values for the sigma though 70 % is a good starting and experimentation can often result in some improvement. To change the sigma value simply type in a new value and then re-train the model (7) to see how this has affected your model output.

Saving Your Model.

Once you have submitted enough training data to your model for you to be happy with the output it is time to save the model. To do this Type in a model name, and optionally a description in the boxes 16 and 17. To save the model click the 'Save Training Model' button (15). The model will then be saved and is accessible for bulk application using the evaluation framework described below. Once training is complete, quit the application by clicking the 'x' button on the application window.

Evaluating Images

For small-scale experiments or testing it is quite possible to just use the model training software. For large bulk datasets it is best to use the evaluation aspect of the QuantiFly software. In this software a number of images are loaded along with a previously trained model. The images are then analysed in bulk. The algorithm is designed to memory efficient so any number of images can be selected and the algorithm will load and process them sequentially.

Running QuantiFly bulk software:

1) Locate the 'QuantiFly-bulk' software which you should have downloaded and unzipped from the website https://github.com/dwaithe/quantifly

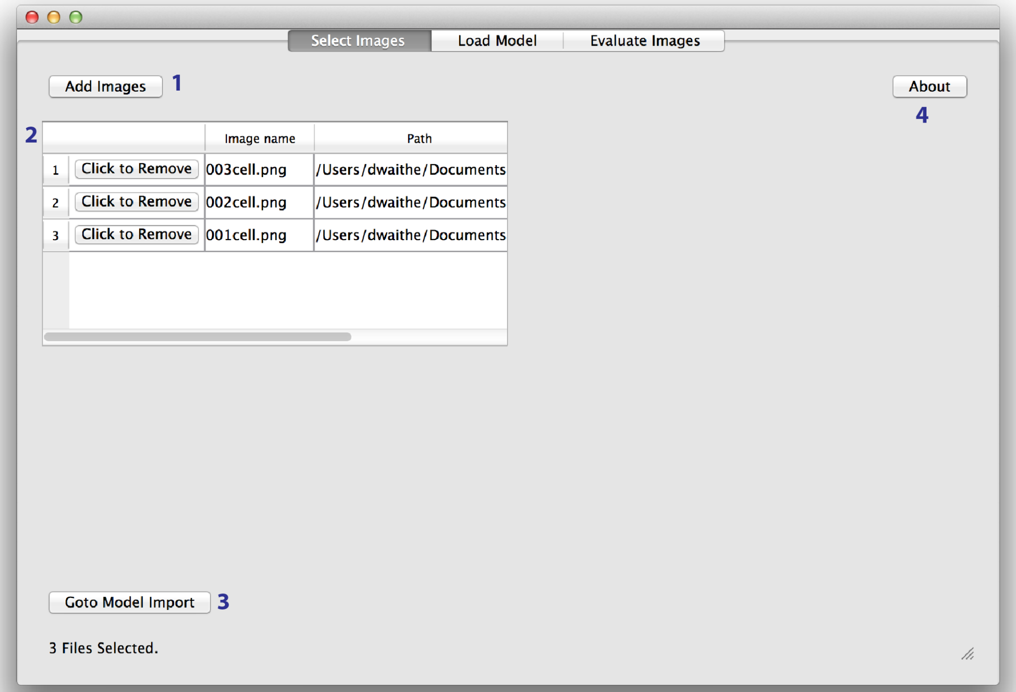

1) Upon loading of the software, the 'Select Image' pane is loaded. See the below figure. To select images for evaluation click the 'Add Images' button (1). The selected images will then be shown in the table which appears below (2). Further images can be added to this list by again using the 'Add Images' button. Each selected image can be removed by clicking the 'Click to Remove' button positioned to the left of each entry in the table.
2) Once you are happy with the list of selected images proceed to the model selection screen by using the Goto Model Import button (3). To view the license agreement for using QuantiFly please click 4.
3) In the 'model import' section of the software a list of all the saved models which are saved on the system are displayed, see in below figure. The name of each model is displayed in the table along with the date it was saved(1). By clicking the 'Click to View' button it is possible to view details of the model. The model preview (2) area shows a representative image, the name, description and some of the parameters of the model.
4) Once you have found the model which you want to use to evaluate your images, make sure you have clicked the 'click to View' button and then click the button marked: 'Goto Image Evaluation' (3).

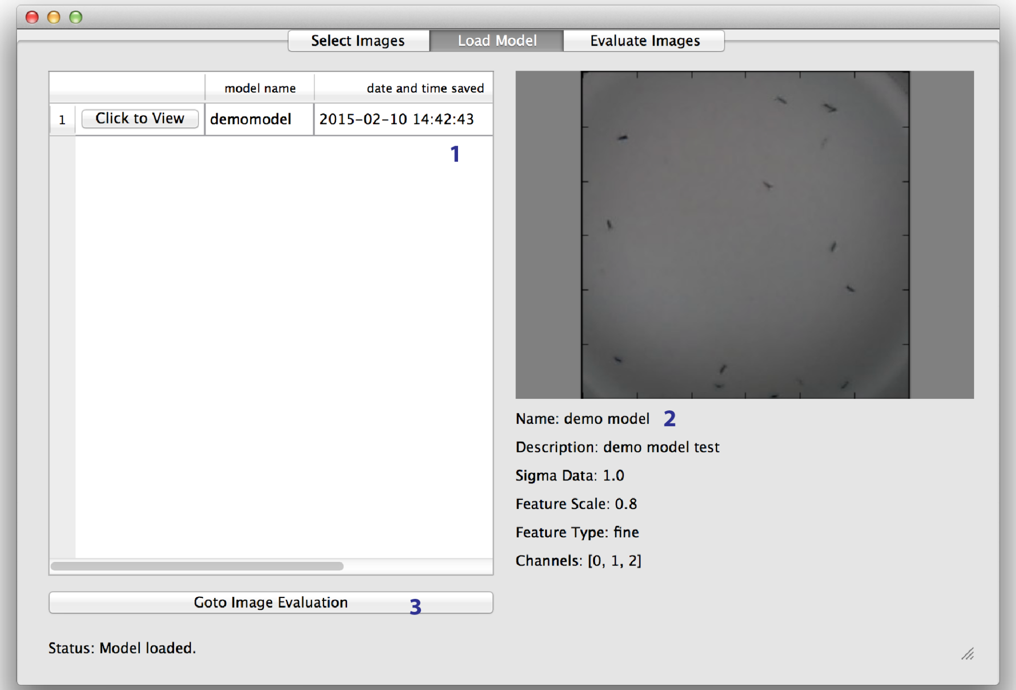

5) Using 'Evaluate Images' tab it is possible to evaluate your images using your chosen model and to output your results, see below figure. You will be able to navigate through your images using the 'Prev Image' and 'Next Image' buttons (1). You can switch channels 'on' on 'off' with the check boxes located at 2. You can see the current image number using the dialog located towards the top of the screen (3).
6) To evaluate your images click the 'Evaluate Images' button and then wait for the processing and evaluation to take place (4). The predicted count for an image is shown at 6 and the predicted count with bias correction is shown next to it at 7. The bias correction also comes with a confidence interval (CI). This interval gives a measure of the predictive accuracy of the bias correction, the smaller the error the more sure the algorithm is of the displayed result. For bias correction at least three regions must be highlighted and the eggs within labelled. Once enough images have been labelled the bias correction and CI will automatically appear. Once the images are processed it is possible to save your results to file by clicking the 'Save Output Data' button (5).

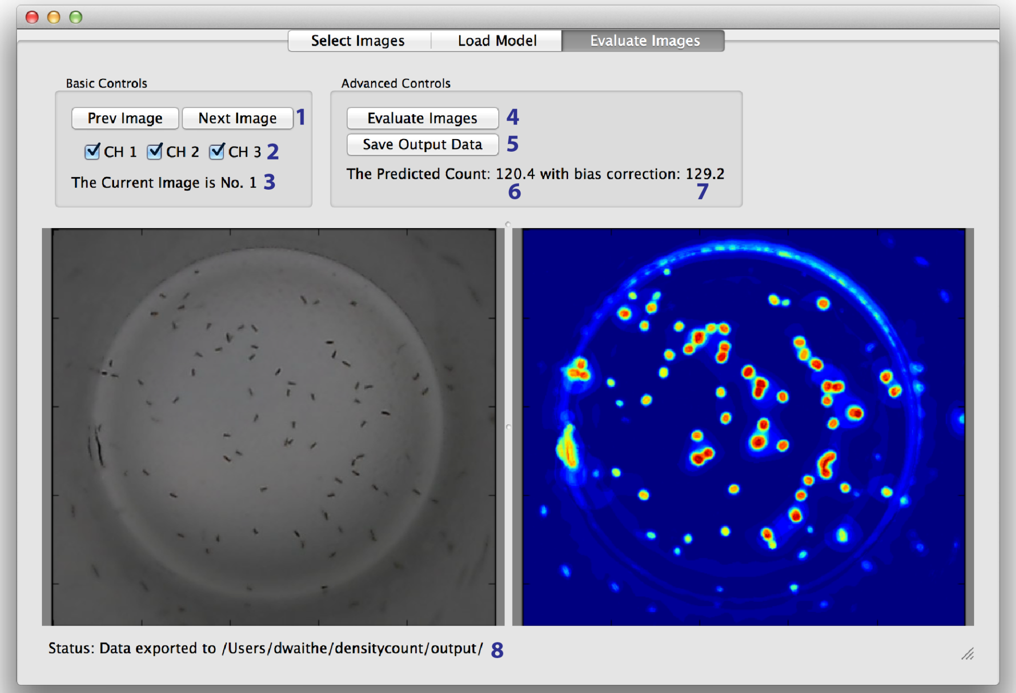

7) Once the evaluation is complete the data is saved to a spreadsheet in the folder shown at (8). The spreadsheet is called output.csv, and can be opened using any conventional spreadsheet software. New data is appended to the end of the spreadsheet. Data is not over-written but time-stamped so it can be identified and the image and model name referenced alongside. Furthermore, in the same folder you can find the output images of the algorithm. These file are saved as 32-bit Tiff images and can be opened in conventional imaging processing software (e.g. Fiji software).

Conclusion

The above document outlines all the steps required in using the QuantiFly software to learn the appearance of object instances in images and how to evaluate further images in bulk. This approach to the counting problem can be used for many different imaging problems and this software makes the application of this technique straight-forward and intuitive.

References

[2] Victor Lempitsky and Andrew Zisserman. Learning to count objects in images. 2010.

[1] Luca Fiaschi, Ullrich Koethe, Rahul Nair, and Fred A Hamprecht. Learning to count with regression forest and structured labels. In Pattern Recognition (ICPR), 2012 21st International Conference on, pages 2685{2688. IEEE, 2012.
